# Alginate gels crosslinked with chitosan oligomers – a systematic investigation into alginate block structure and chitosan oligomer interaction†

**DOI:** 10.1039/d1ra01003d

**Published:** 2021-04-13

**Authors:** Georg Kopplin, Anders Lervik, Kurt I. Draget, Finn L. Aachmann

**Affiliations:** Norwegian Biopolymer Laboratory (NOBIPOL), Department of Biotechnology, Norwegian University of Science and Technology 7491 Trondheim Norway finn.l.aachmann@ntnu.no; Department of Chemistry, Norwegian University of Science and Technology 7491 Trondheim Norway

## Abstract

Three alginates with fundamentally different block structures, poly-M, poly-G, and poly-MG, have been investigated upon ionic crosslinking with chitosan oligosaccharides (CHOS), using circular dichroism (CD), rheology, and computer simulations, supporting the previously proposed gelling principle of poly-M forming zipper-like junction zones with chitosan (match in charge distance along the two polyelectrolytes) and revealing a unique high gel strength poly-MG chitosan gelling system. CD spectroscopy revealed an increased chiroptical activity exclusively for the poly-M chitosan gelling system, indicative of induced conformational changes and higher ordered structures. Rheological measurement revealed gel strengths (*G*′ < 900 Pa) for poly-MG (1%) CHOS (0.3%) hydrogels, magnitudes of order greater than displayed by its poly-M analogue. Furthermore, the ionically crosslinked poly-MG chitosan hydrogel increased in gel strength upon the addition of salt (*G*′ < 1600 at 50 mM NaCl), suggesting a stabilization of the junction zones through hydrophobic interactions and/or a phase separation. Molecular dynamics simulations have been used to further investigate these findings, comparing interaction energies, charge distances and chain alignments. These alginates are displaying high gel strengths, are known to be fully biocompatible and have revealed a broad range of tolerance to salt concentrations present in biological systems, proving high relevance for biomedical applications.

## Abstract 2

Three alginates with different block structures, poly-M, poly-G, and poly-MG, were investigated upon ionic crosslinking with chitosan oligomers, using circular dichroism, rheology, and simulations, revealing a unique high gel strength poly-MG chitosan gelling system.

## Graphical abstract

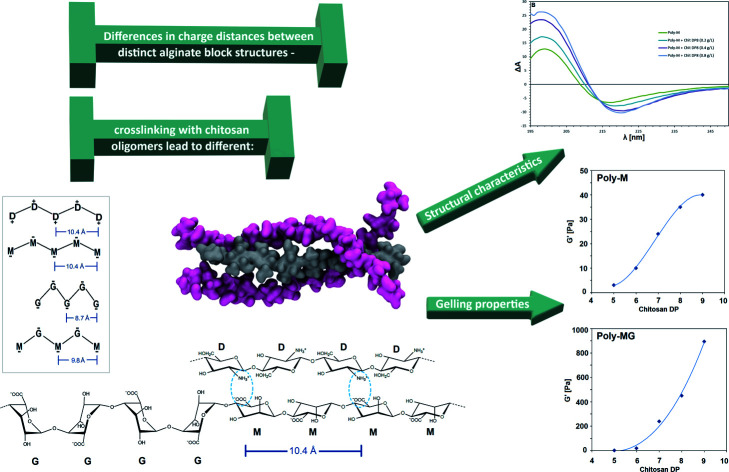

## 1 Introduction

Alginate is a linear polysaccharide predominantly occurring in nature in the class of brown seaweed *Phaeophyta*,^1^ although various bacteria such as *Pseudomonas* and *Azotobacter vinelandii* incorporate alginate as exocellular polymeric material.^2,3^ Alginates form a polysaccharide family composed of (1 → 4)-linked β-d-mannuronic acid (M-unit) in ^4^C_1_ conformation and its C5-epimer, α-l-guluronic acid (G-unit) in the ^1^C_4_ conformation with a p*K*_a_-value of circa 3.5. These unbranched polyanionic block copolymers are composed of homopolymeric regions of M-units (M-blocks), G-units (G-blocks), and regions of alternating epimers (MG-blocks) of various lengths.^4,5^

Chitosans form a family of linear polysaccharides consisting of (1 → 4)-β-linked 2-acetamido-2-deoxy-d-glucopyranose (GlcNAc) and its de-*N*-acetylated analogue (GlcN). These polycationic derivatives of chitin can be prepared with varying degrees of acetylation (*F*_A_) and polymerization (DP).^6^ The amine-group of the D-unit (GlcN) has a p*K*_a_-value of circa 6.5,^7–9^ and therefore their water-solubility correlates with their *F*_A_ and the surrounding pH.^10^

Oligomer fragments of these polysaccharides, referred to as CHito-OligoSaccharides (CHOS), can be prepared using chemical or enzymatic methods. CHOS have attracted increasing attention in recent years as they have been associated with numerous biological effects.^11–14^ Chitosan is known for its high biocompatibility, biodegradability^15,16^ and low toxicity.^17,18^

Moreover, antimicrobial effects have been shown,^19–22^ which are relevant for applications such as drug delivery, wound healing and implants.^23–25^ For the development of novel applications, chitosans with a tailored DP, polydispersity, F_A_ and acetyl distribution are providing another tool for influencing the functions, physical properties and biological effects of biomaterials.

Hydrogels are networks of cross-linked hydrophilic polymer chains able to retain large amounts of water. They are defined by specific physical properties regarding their dynamic moduli, describing elastic and viscous response to mechanical force.^26^ Cross-links within hydrogels can be established through hydrogen bonds^27^ and hydrophobic interactions.^28,29^ Ionically crosslinked hydrogels are stronger affected by changes in pH and exhibit a higher sensitivity towards swelling than their covalently crosslinked counterparts. These different properties broaden their spectrum of applications since further adjustments to their specific environment are possible.^30^

The most common ionically crosslinked alginate gels are established by the addition or release of multivalent cations (*e.g.* Ca^2+^, Sr^2+^ and Ba^2+^). Calcium–alginate hydrogels are intensively studied and exhibit a broad utilization for cell immobilization and the protection of cells from the host's immune system.^31–34^ These ionically cross-linked gels can be prepared through a diffusion method or internal gelation.^4^ The internal gelation method is carried out by mixing insoluble calcium carbonate into the alginate solution, followed by a controlled decrease of the pH through a slow proton-donator such as the d-glucono-δ-lactone (GDL), which results in the homogeneous release of calcium ions and a subsequent gel formation. The calcium ions show specific interactions with the G-units and create junction zones between G-blocks (egg-box formation) and partially MG-blocks.^35^

As an alternative to the formation of junction zones between the G-blocks with divalent cations, cross-linking consecutive M-units of polymannuronic acid with fully deacetylated chitosan oligomers has been previously investigated and the gelling concept, gel strength, kinetics and swelling properties have been characterized.^36,37^ These junction zones are stabilized by a match in charge distance between chitosan and poly-M (^4^C_1_ conformation, diequatorial glycosidic linkage) which are both at 10.4 Å (ref. 38 and ^39^) whereas the G-blocks (^1^C_4_ conformation, diaxial glycosidic linkage) exhibit a shorter charge distance of only 8.7 Å (ref. 40) and do not form (strong) gels with chitosan.

To gain further insight into this new gelling concept, we isolated fully de-*N*-acetylated chitosan oligomers of different DP and systematically applied these CHOS as crosslinker to highly purified and characterized poly-M, poly-G and strictly alternating poly-MG (*F*_G_ = 0.47, *F*_GG_ = 0) alginate.^41^

Through rheological methods detailed information about gel strength and gelling kinetics have been obtained. During this characterization, it has been discovered that while poly-G does not form gels at all, poly-MG is able to form up to 10-fold stronger gels than its poly-M analogue.

This counterintuitive phenomenon that the strongest hydrogel is formed by an alginate deviating in charge distance from its crosslinker chitosan, has been further investigated through circular dichroism in order to determine potential structures of higher order or conformational changes within the alginate molecule,^42^ as well as through molecular dynamics simulations to calculate differences in interaction strengths, ionic bond lengths and alignments, ruling out different explanations for this peculiar phenomenon and refining the previously presented gelling concept.

## 2 Experimental

### 2.1 Materials

#### Alginates

High molecular weight mannuronan (poly-M) was isolated from a C-5 epimerase negative mutant of *Pseudomonas fluorescens* according to Holtan (2006).^43^ Poly-MG was synthesized by epimerizing a poly-M alginate using AlgE4 epimerase as previously described.^44^ The enzyme processively epimerizes every second mannuronic acid residue into guluronic acid, converting the substrate almost entirely into a strictly polyalternating MG-sequence (*F*_G_ = 0.47, *F*_GG_ = 0).^41^ A guluronic acid enriched alginate (poly-G) was prepared by *in vitro* epimerization with recombinantly produced C-5 epimerase AlgE6 as previously described.^45^ The intrinsic viscosity of all 3 alginates was determined using an Ubbelohde capillary viscometer (Schott-Geräte) equipped with an AVS 310 control unit and PC-operated titrator. Nine concentrations (10 to 0.5 g L^−1^, solvent 0.1 M NaCl) were analysed at 20.0 °C with 4 repetitions each. Automatic data acquisition and calculations was used, applying a Mead–Fouss, Huggins, Billmeyer, and Herman plot.

The molecular weight average was measured by Size-Exclusion Chromatography (SEC) using an HPLC system fitted with online Multi-Angle static Light Scattering (MALS) Dawn HELEOS-II multi-angle laser light scattering photometer (Wyatt, U.S.A.) (*λ*_0_ = 663.8 nm) followed by an Optilab T-rEX differential refractometer. The eluent was 0.15 M NaNO_3_/0.01 M EDTA (pH = 6, I = 0.17 M) and the flow rate was 0.5 mL min^−1^. The data were collected and processed using the Astra (v. 6.1) software (Wyatt, U.S.A.).

#### Chitosan oligomers

Fully de-*N*-acetylated chitosan (*F*_A_ = 0.001) was prepared by a further heterogeneous de-*N*-acetylation of a commercially available chitosan (Kimica, Japan). Fully de-*N*-acetylated chitosan oligomers were prepared by enzymatic hydrolysis of the fully de-*N*-acetylated chitosan using a commercial chitosanase from *Streptomyces griseus* (Sigma C9830) and a subsequent separation using size exclusion chromatography.^39^

In addition a chitosan oligomer mixture (DP_*n*_ = 3.96, *F*_A_ = 0.045) provided by Koyo Ltd Co (Japan), lot number 121017WG was used in comparison.^37^d-Glucono δ-lactone (GDL) was purchased from Sigma-Aldrich (U.S.A.). All other chemicals were of analytical grade and used without any further purification.

### 2.2 Gel preparation

The different alginates were dissolved in distilled water at a concentration of 3% (30 g L^−1^). The selected chitosan oligomers were dissolved in distilled water at a concentration of 10% and the pH was adjusted to 8.0, using 1 M NaOH. 1.0 mL of 3% alginate solution was weighted into a glass vial and a maximum of 182 μL of the selected chitosan oligomer solution (equaling 0.6% final concentration, see ESI†) was added together with distilled water to obtain a total volume of 2 mL. The mixture was stirred for 10 minutes. 15.95 mg of GDL were solved in 1 mL distilled water right before adding to mixture (final alginate concentration of 1%) followed by intensive stirring for 30 s and immediate casting on the rheometer bottom plate.

### 2.3 Rheological measurements

Rheological measurements were performed with a Kinexus rheometer (Malvern Instruments, United Kingdom), using a 40 mm serrated plate probe and a serrated bottom plate with a 1 mm gap.

The test methods employed were oscillatory time and stress sweep at a constant temperature of 20 °C. For the time sweep, the experiments were performed at a low oscillation frequency (1 Hz) and a shear strain (0.001) to ensure that the measuring conditions did not disrupt the gelation process. The strain sweep (0.0005 to 0.1), at a constant frequency of 1 Hz, was used to ensure the strain lays in the linear viscoelastic region (LVR) of the hydrogels. Frequency sweep experiments were performed in the linear viscoelastic region (0.01 to 10 Hz) with a constant strain of 0.001 and a delay time of 2 seconds between the measurements to characterize the viscoelastic properties as a function of frequency. Three parallels were performed for each experiment.

### 2.4 Circular dichroism

Circular dichroism spectra were recorded using a Chirascan™ V100 equipped with a Chirascan™ monochromator using two synthetic, single-crystal quartz prisms. All samples were recorded in distilled water at 25 °C. A quartz cuvette with 5 mm optical pathlength was used and an array from 180 to 260 nm was scanned with a bandwidth of 1 nm, a time constant of 3 s (scan rate 20 nm min^−1^). The stock solutions of the different alginates and chitosan oligomers were prepared short before the experiments and adjusted to pH 4.5 to ensure strong interaction between the chitosan oligomers and the alginate.^37^ The final concentration of alginate was kept constant at 0.4 g L^−1^, the chitosan oligomer concentration was systematically increased from 0.1 to 0.8 g L^−1^.

### 2.5 Molecular dynamics simulations

Molecular dynamics (MD) simulations were carried out using GROMACS (version 5.1.4).^46^ Initial configurations, consisting of the different types of alginates and chitosan tetramers or octamers, were created using the 3D builder tool in Maestro.^47^ These initial geometries were optimized at the B3LYP-D3/6-31** level of theory using Jaguar,^48^ and the different configurations we considered are detailed in Section 3.6. After the geometry optimization, the configurations were converted to GROMACS, and the OPLS-AA force field was used to model the interactions.^49^ Coulombic forces were obtained using the smooth particle mesh Ewald (PME) method,^50^ van der Waals interactions were truncated at 1.4 nm, and dispersion corrections were applied to the energy and pressure terms. Further, periodic boundary conditions were applied in all directions, and all bonds were constrained using the LINCS algorithm.^51^ Following a geometry optimization using a steepest descent algorithm as implemented in GROMACS, the alginate-chitosan molecules were solvated by adding ions (Na^+^ and Cl^−^) and water molecules using the TIP4P model of water,^52^ in agreement with experimental concentrations and electroneutrality. To equilibrate the solvated system, a short (100 ps) MD simulation was carried out using the thermostat of Bussi *et al.* (2007) (with a target temperature of 300 K and a coupling parameter of 0.1 ps) and an isotropic Berendsen barostat (with a target pressure of 1 bar, coupling parameter of 2 ps and isothermal compressibility of 4.5 × 10^5^ bar^−1^).^53,54^ Production MD simulations were then carried out in the isothermal–isobaric ensemble where the temperature was controlled using the same thermostat as detailed above, and the pressure was controlled with an isotropic Parrinello–Rahman barostat (same settings as given above).^55^ The equations of motion were integrated using a Leap–Frog integrator with a time step of 2 fs and the production simulations were carried out for 100–350 ns in which we monitored the interactions and the configurations.

## 3 Results

### 3.1 Characterization of alginates

The three different types of alginate of poly-M, poly-G and poly-MG used in this study, were selected based on their guluronic acid (G) and mannuronic acid (M) composition as well as their diad fraction *F*_GG_, *F*_MG_ and *F*_MM_ (see Table 1). Monad and diad composition were determined using NMR spectroscopy according to Grasdalen *et al.* (1981, 1983), revealing three alginates with different almost exclusively homopolymeric block-structures.^56,57^ Additionally, the three samples were selected based on their molecular weight average (*M*_w_) and intrinsic viscosity ([*η*]), to mitigate the influence of their molecular size on the conducted gelling experiments.^58,59^

**Table 1:** Characterization of alginates through their monad and diad fraction, their molecular weight and number average and their intrinsic viscosity

Alginate	*F*_G_	*F*_M_	*F*_GG_	*F*_GM_	*F*_MM_	*M*_w_ [kDa]	*M*_n_ [kDa]	[*η*] [mL g^−1^]
Poly-M	0.00	1.00	0.00	0.00	1.00	232	104	740
Poly-MG	0.47	0.53	0.00	0.47	0.07	158	65	534
Poly-G	0.88	0.12	0.83	0.05	0.07	225	105	727

### 3.2 Characterization of chitosan oligomers

**Fig. 1:**
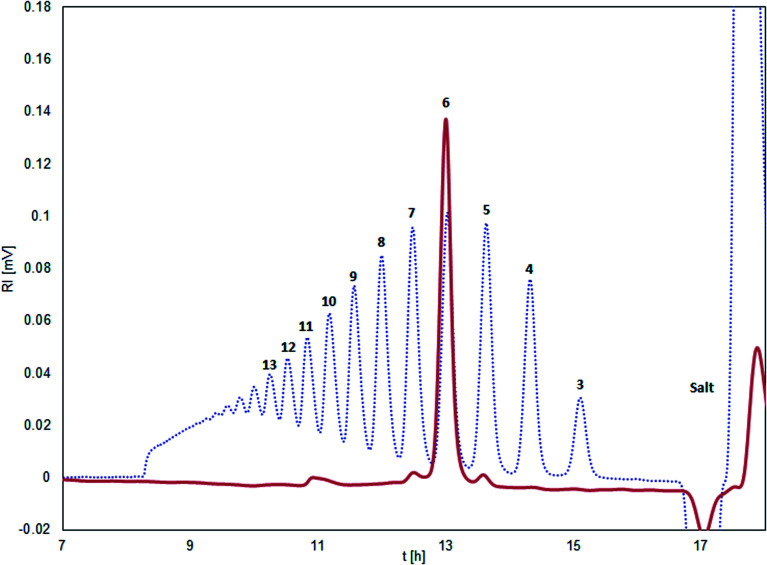
Size exclusion chromatogram of chitosan (*F*_A_ = 0.001) degraded by chitinase from *Streptomyces griseus* (blue dotted line). Isolated chitosan hexamer (red line). The number of glucosamine units in the oligomer peak is given in bold numbers.

The isolated chitosan oligomers through SEC-fractionation, showed a purity of ≥95% with only minor impurities from the sequentially neighboring oligomers DP_*n*±1_ (see Fig. 1).

### 3.3 Gel strength and kinetics of gelation as function of chitosan oligomer length

**Fig. 2:**
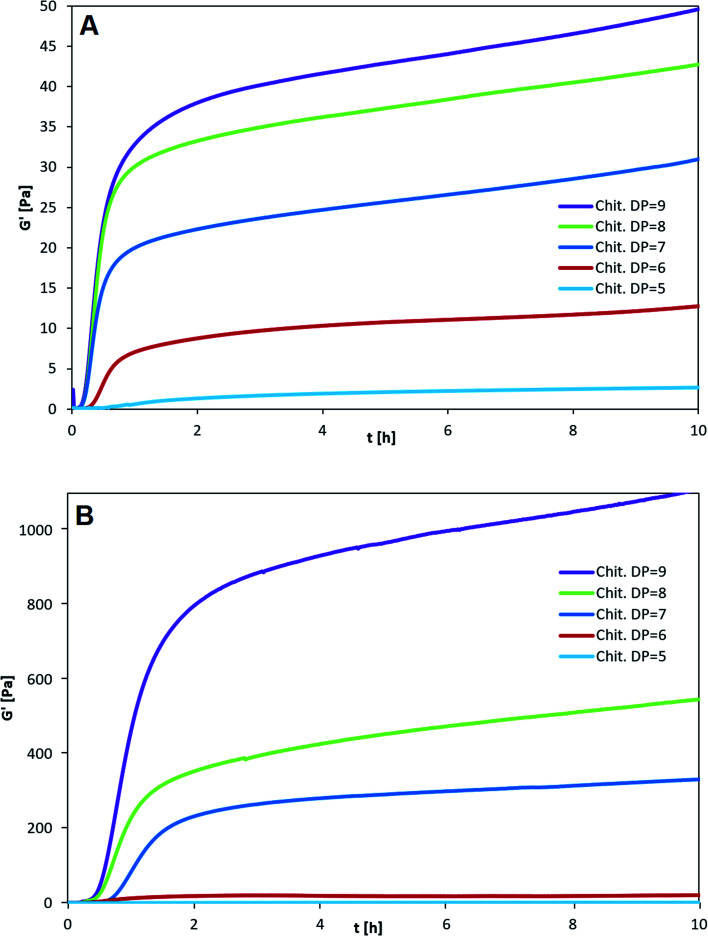
Influence of NaCl concentration on the kinetics of gelation for (A) poly-M and (B) poly-MG. Alginate concentration at 10 g L^−1^ (1%), chitosan octamer concentration at 3 g L^−1^ (0.3%), GDL at 2 g L^−1^, NaCl concentration added at 0 mM (red), 50 mM (blue), 150 mM (green), 500 mM (purple). *G*′ was determined as a function of time. *G*′′ and delta are not shown in the figure for reasons of clarity.

The three types of homogeneous block-polymers poly-M, poly-MG and poly-G were tested against a set of purified chitosan oligosaccharides (CHOS), consecutively increasing in degree of polymerization from DP = 5 to 9 (see Fig. 2). The kinetics of gelation were followed by measuring the storage modulus (*G*′) and the loss modulus (*G*′′) as a function of time (*t*). The CHOS concentration (w/v%) was identical for each setup and the pH was monitored, resulting in a final pH of 4.35 ± 0.05 (measured after 5 h). This pH has previously been found to give the strongest CHOS-alginate gels while simultaneously showing small pH related deviations in gel strength.^37^

In agreement with previously reported data, poly-G displayed no signs of gel formation for any chitosan oligomer independent of DP and concentration.^36^ Through rheological measurements of poly-G chitosan oligomer gelling systems high phase angles (*δ*) close to 90° were obtained, representative of a viscous solution (see ESI†). An increase in chitosan oligomer concentration to half of the poly-G concentration (or higher), lead to a precipitation of the alginate, likely caused by non-specific electrostatic interactions.

When comparing poly-M with poly-MG the mannuronic acid homopolymer showed faster gelling kinetics, reaching an apparent equilibrium after about 20–30 min for all different chitosan DPs (Fig. 2A), while the poly-MG gelling system reached the apparent equilibrium after approximately 60–90 min (Fig. 2B).

For all CHOS-poly-M gelling systems, the phase transition from predominant viscous (*G*′ < *G*′′) to predominant elastic (*G*′ > *G*′′) properties, where the phase angle drops below 45°, occurred within the first minute, which indicates a fast gelling system (*G*′′ and *δ* are not depicted here). For poly-MG the phase transition occurred after about 5 minutes for DP = 6–9 and after 30 min for DP = 5.

**Fig. 3:**
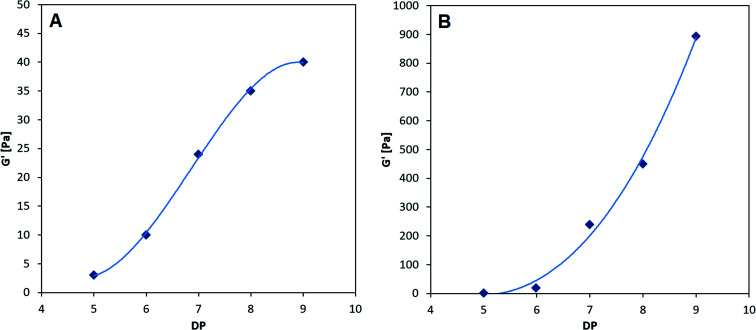
Final gel strength as a function of degree of polymerization of chitosan. (A) Poly-M; (B) poly-MG. Alginate concentration at 10 g L^−1^ (1%). Chitosan concentration at 3 g L^−1^ (0.3%). GDL at 2 g L^−1^. *G*′ value determined after 5 h.

The smallest CHOS capable of forming poly-M gelling systems is the fully de-*N*-acetylated chitosan tetramer.^37^ However, poly-MG was not capable of forming a gel with chitosan at DP = 4, neither with a pure tetramer nor with a commercial chitosan oligomer mixture (DP_*n*_ = 3.96) (see ESI†). For DP = 4 the viscoelastic properties of poly-MG are mimicking the ones of poly-G, resulting in a precipitation of the alginate instead of a gel formation. CHOS at DP = 5 are the shortest fully de-*N*-acetylated chitosan oligomers capable of establishing a poly-MG hydrogel. Those poly-MG chitosan gels at DP = 5 expressing a lower *G*′ than their poly-M counterpart. While the chitosan hexamer displays in a similar *G*′ value in poly-M and poly-MG systems. For CHOS with a DP ≥ 7 poly-MG expresses a sharp increase in gel strength, displaying a storage modulus orders of magnitude greater than for poly-M. Furthermore, a different trend for both types of alginate can be observed. While an increase in chitosan oligomer DP leads to an increase in gel strength in both types of alginate, the relative increase between consecutive oligomers becomes smaller in poly-M systems (see Fig. 3). In poly-MG systems on the other hand, the formed gels exhibit a strong increase in the storage modulus towards DP = 9, both in absolute and relative amounts.

Alginate-CHOS gelling systems with chitosan oligomer DPs ≥ 10 have been prepared, but the formed hydrogels were inconsistent in texture due to a decreasing solubility at pH 8 of the higher chain length CHOS and measurements showed strong deviations.^10,36^

A frequency sweep control experiment of the poly-MG CHOS nonamer system was performed, without adding the proton donator d-glucono-δ-lactone (GDL), showing liquid like properties at pH 7.5 (phase angle close to 90°) along a broad frequency range from 0.01 to 10 Hz (see ESI†). Those findings are in agreement with previously measured poly-M and poly-G systems.^36,37^ To control for temperature related effects on the gel formation potentially caused by an increased or decreased molecular mobility, both poly-M and poly-MG were gelled at 4 °C, 20 °C and 40 °C, using the CHOS octamer. After 5 h the temperature was adjusted to 20 °C where no significant difference in final gel strength between the 3 samples gelled at different temperatures could be observed (data not shown).

### 3.4 Gel strength and kinetics of gelation as function of salt concentration

Chitosan (DP = 8) crosslinked alginates hydrogels made from either poly-M or poly-MG were tested against a set of different salt (NaCl) concentration. The kinetics of gelation were followed by measuring the storage modulus (*G*′) and the loss modulus (*G*′′) as a function of time (*t*). Since both, alginate as well as chitosan oligomers are introduced in their salt form, the formation of each ionic bond between the carboxyl and amino groups will release one NaCl equivalent. At a chitosan oligomer concentration of 3 g L^−1^ this leads to an initial NaCl concentration of maximum 15 mM.

**Fig. 4:**
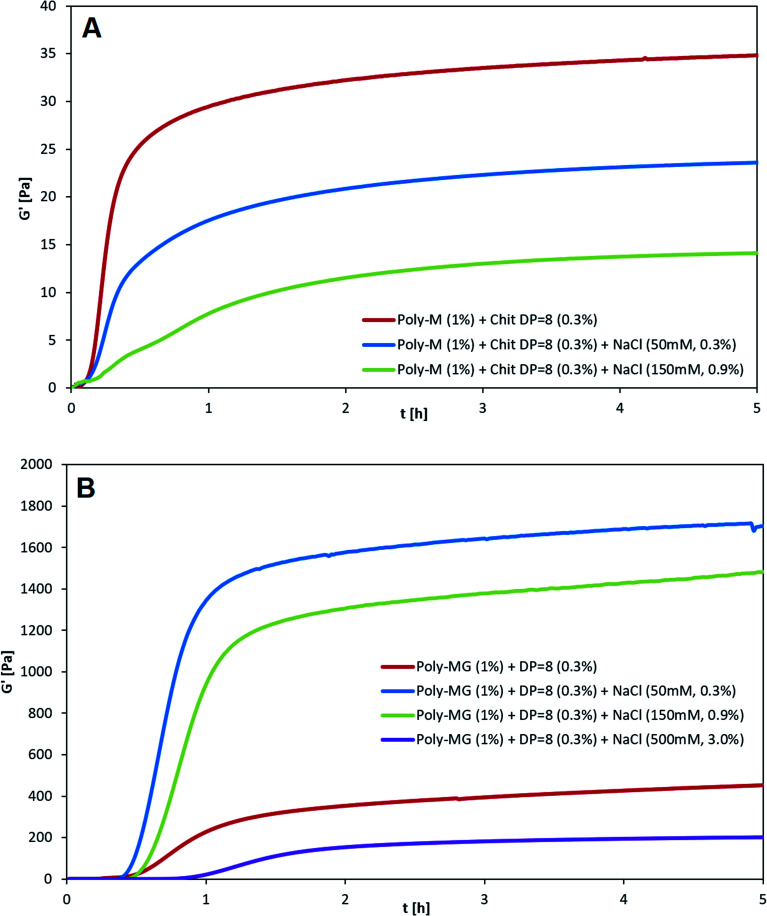
Influence of NaCl concentration on the kinetics of gelation for (A) poly-M and (B) poly-MG. Alginate concentration at 10 g L^−1^ (1%), chitosan octamer concentration at 3 g L^−1^ (0.3%), GDL at 2 g L^−1^, NaCl concentration added at 0 mM (red), 50 mM (blue), 150 mM (green), 500 mM (purple). *G*′ was determined as a function of time. *G*′′ and delta are not shown in the figure for reasons of clarity.

After adding 50 mM NaCl to the chitosan poly-M gelling system, a decrease to two thirds of the original gel strength was observed (see Fig. 4). An addition of 150 mM NaCl decreased the gel strength to one third of the original gel strength. When 500 mM NaCl were added, no stable gel formation was observed any longer. Beside the decrease in gel strength, the gelling kinetics slowed down as well. The apparent equilibrium for the plain mannuronate chitosan sample occurred after 20 minutes, followed by 40 min for the 50 mM NaCl sample and 100 min for the 150 mM NaCl sample. A decrease in both gel strength and gelling kinetics is well described for alginates and other ionically crosslinked hydrogels.^60–63^

For poly-MG crosslinked with the chitosan octamer an almost opposite effect upon the addition of salt was observed. Instead of a decrease, the gel strength quadrupled when 50 mM NaCl was added to the gelling system. Upon an addition of 150 mM NaCl, the storage modulus *G*′ was about three times higher than for the equivalent mixture without added salt. In contrast to the poly-M counterpart, the poly-MG chitosan system could form a stable gel upon the addition of 500 mM NaCl. The obtained gel strength for this high salinity mixture was reduced to about half of the original poly-MG chitosan octamer gelling system. The gelling kinetics for the poly-MG CHOS gelling systems showed to be almost not effected upon an increase in salt concentration, except for the highest salt concentration at 500 mM which displayed a decrease in gelling kinetics. The apparent equilibria occurred after 50, 60, 70 and 130 min for 0, 50, 150 and 500 mM of added NaCl, respectively.

### 3.5 Circular dichroism

**Fig. 5:**
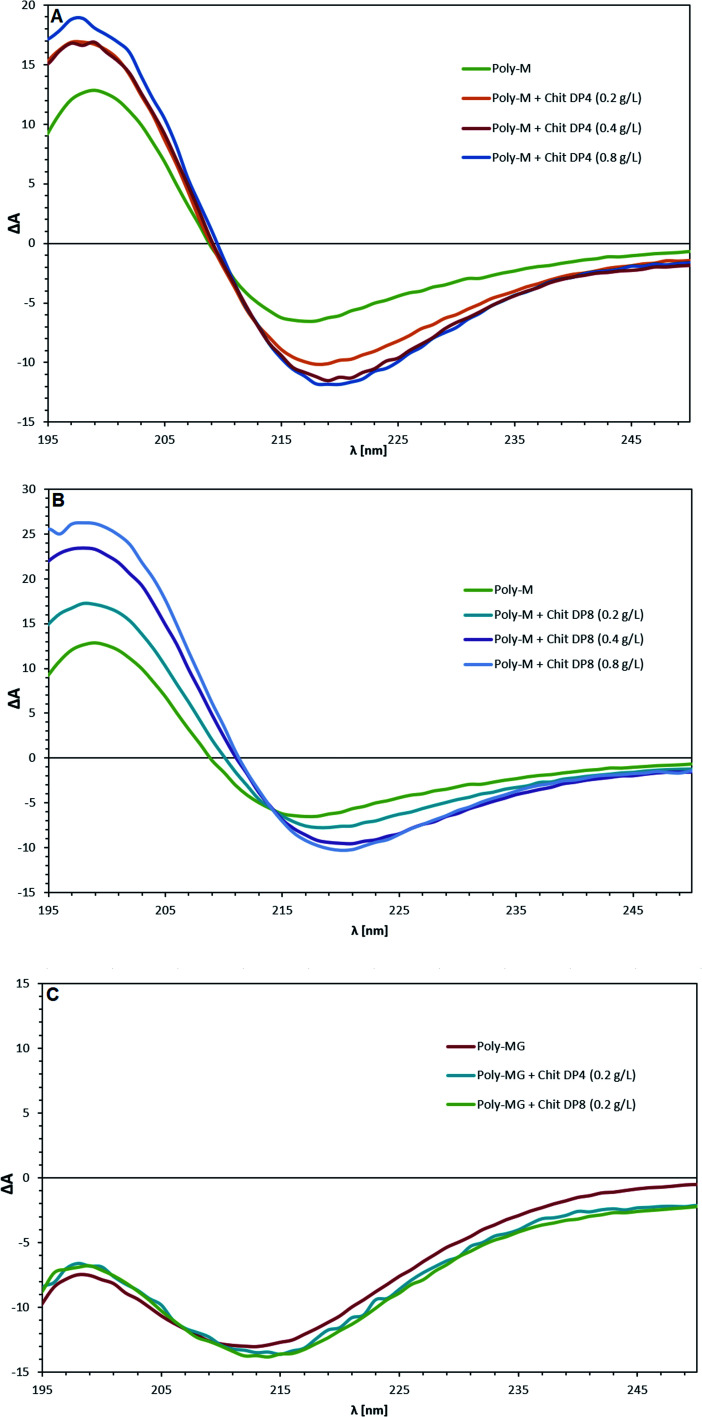
Circular dichroism spectra of (A) poly-M with chitosan DP = 4; (B) poly-M with chitosan DP = 8; and (C) poly-MG with chitosan DP = 4 and DP = 8. The alginate concentration was consistently at 0.4 g L^−1^. The chitosan concentration ranged from 0.2 to 0.8 g L^−1^. All measurements performed at pH = 4.5 and *T* = 25 °C.

The pure poly-M, poly-MG and poly-G alginate samples were analyzed through circular dichroism and spectral changes upon the addition of chitosan oligomers were investigated. The CD spectra of the three pure types of alginate are in agreement with previously reported data.^64^ The poly-G lies completely in the area of negative ellipticity, while poly-M shows an intense positive band between 190–210 nm, originating from n→π* transitions (with minor contributions of π→π* transitions below 200 nm).^65^ The CD spectrum of alternating MG-sequences, lies in between although is not identical to an equimolar mixture of poly-M and poly-G (see ESI†).^64,66^ By addition of chitosan oligomers with a degree of polymerization of 4 to poly-M alginate (Fig. 5A), the positive band between 190–210 nm shows a strong increase while the trough at 210–240 nm is deepened, suggesting an alignment of chitosan oligomers and poly-M alginate into a higher ordered conformation.^67^ An increase in chitosan tetramer concentration from 0.5 to 1.0 to 2.0 times the alginate concentration (based on monosaccharide unit concentration, see ESI†) does not increase this effect. The bands only increase for the amount that chitosan itself adds to the cumulative measurement value (see ESI†). By addition of chitosan octamer, the effect at half-concentration towards the alginate was almost identical to the effect observed when using the DP = 4 oligomer but increased further at equal concentration of chitosan and alginate. Double-concentration of DP = 8 showed an even further increase of this effect, which was higher than the cumulative contribution to the CD band by the chitosan itself. The peak maxima for all poly-M-DP = 8 setups can be found at 199 nm. The trough minima are located at 220 nm (and 217 nm for pure poly-M, respectively). A cross section for all parallels was found at 214 nm.

The CD spectrum of poly-MG displayed no change upon addition of both chitosan DP = 4 and DP = 8 at half-concentration of the alginate. Equal concentration lead to a precipitation and concealed the alginate bands. Poly-G (see ESI†) followed the same trend as poly-MG, exhibiting no CD change induced through chitosan oligomers at half-concentration. An equal concentration of chitosan and poly-MG lead to precipitation of the aggregate.

### 3.6 Molecular dynamics simulation

Different sets of alginate dodecamers consisting of only M-, G- or strictly alternating MG-units were simulated interacting with chitosan tetra- or octamers. The chitosan and alginate oligomers, their concentration and ratio to each other were varied, as well as the present salt concentration. Water molecules were added to the system to make the concentration of alginate equal to 0.05 M. For the salt concentration, two different cases were considered: a low concentration system where ions (Na^+^ and Cl^−^) were added to make the whole system electroneutral and a high concentration system where ions were added to match the experimental concentrations.

**Fig. 6:**
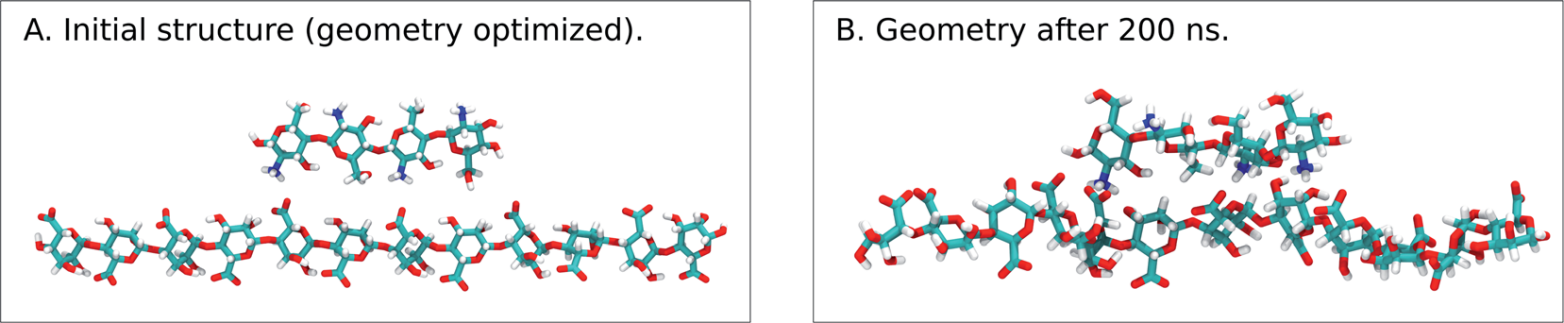
Snapshots from a MD simulation of the MG-oligomer (DP = 12) and the chitosan oligomer (DP = 4) (Na^+^ and Cl^−^ ions are not shown). (Left) The initial geometry optimized configuration. (Right) The configuration after a MD simulation lasting 200 ns, showing some distortions compared to the initial structure.

**Fig. 7:**
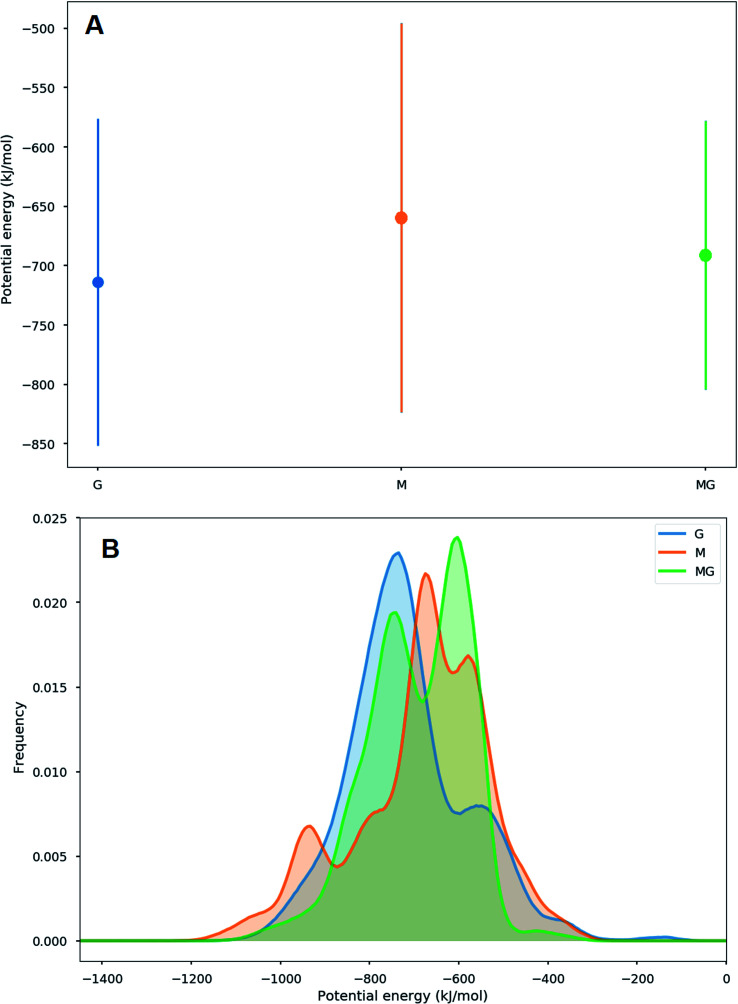
Average (A) and histograms (B) of the interaction energies between alginate oligomers (DP = 12) and a chitosan oligomer (DP = 4). The average interaction energy is lowest for the G-oligomer and largest for the M oligomer, however there are significant overlaps between the distributions of the interaction energies in this case. These simulations were carried out for 200 ns for systems with a low concentration of salt. A more negative potential energy corresponds to a stronger interaction.

When simulating one M-oligomer, one G-oligomer, or one MG-oligomer (all with DP = 12) interacting with chitosan of DP = 4, no significant difference in molecular distance or alignment emerged. Histograms showing the interaction potential energies between the alginate and chitosan are shown in Fig. 7 and snapshots from the simulation of the MG-oligomer are shown in Fig. 6. In all these simulations, the alginate oligomer and the chitosan oligomer remain bounded.

When applying a chitosan octamer to a M-, G-, or alternating MG-dodecamer in 1 : 1 ratio, only small differences were revealed. However, within the margin of error, the interaction of chitosan with the G-oligomer appears to be slightly stronger. The interactions between the alginate and the chitosan as well as simulation snapshots are shown in the ESI.†

**Fig. 8:**
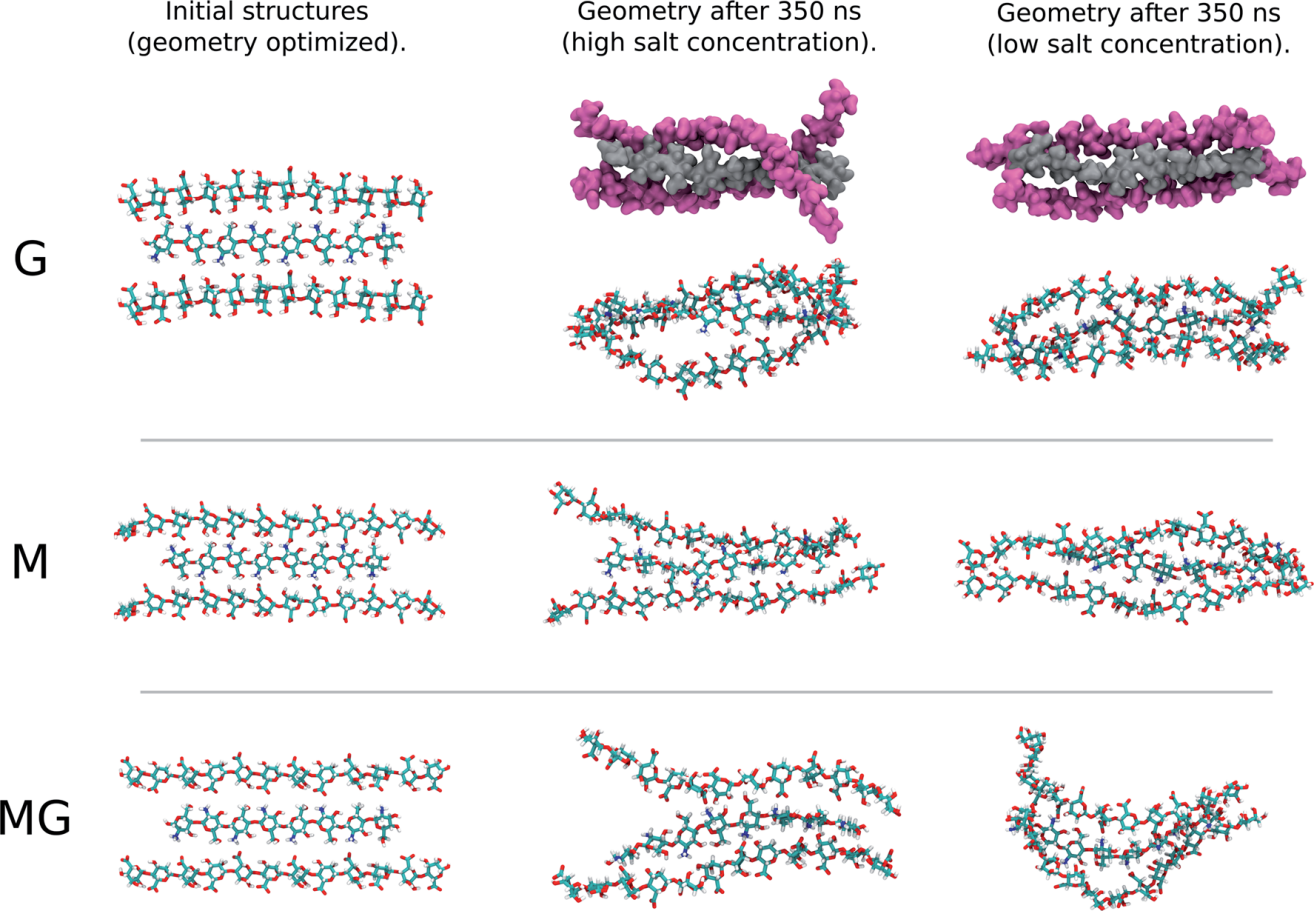
Snapshots of structures from MD simulations of the 2 : 1 alginate : chitosan systems. For the G-oligomer, space filling structures are also depicted to exemplify how the alginate oligomers (color pink) can wrap around the chitosan oligomer (color gray).

**Fig. 9:**
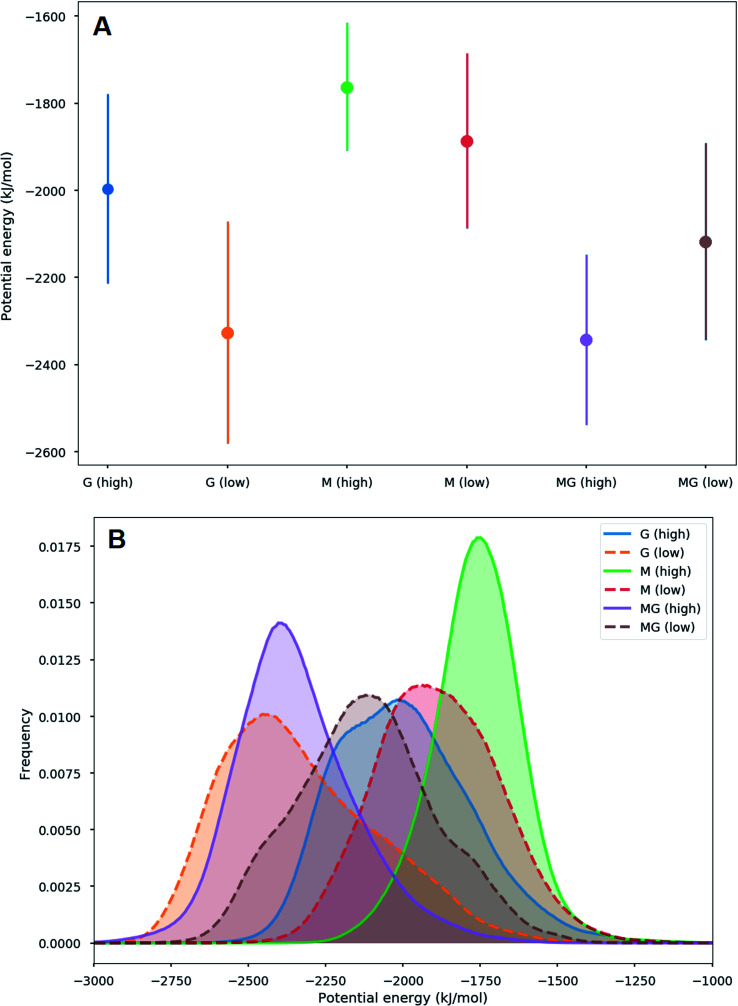
Averages (A) and histograms (B) of interaction energies for the 2 : 1 alginate : chitosan systems considered, obtained in MD simulations lasting 350 ns. The average interaction energy is lowest for the MG-alginate system with a high salt concentration, while it is largest for the M-alginate system with a high salt concentration.

**Fig. 10:**
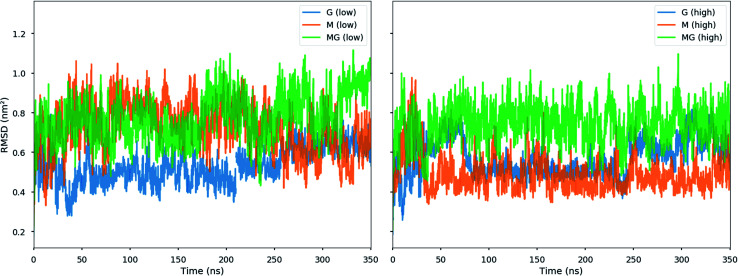
Root mean square deviation (RMSD) from MD simulations of the 2 : 1 alginate : chitosan systems. The RMSD is calculated with respect to the original geometry optimized structures in vacuum and shown for the low salt concentration (left) and the high salt concentration (right).

The simulations of alginate and chitosan oligomers on a 2 : 1 ratio, closer to the conditions in the macroscopic gelling system, where junction zones are formed between two alginate segments ionically crosslinking through chitosan, revealed that the strongest interaction between chitosan and alginate occurred for the MG-oligomers with a high salt concentration. This was closely followed by the G-alginate (with a low salt concentration), which in macroscopic systems precipitates upon the addition of chitosan oligomers.^36^Fig. 9 shows the interaction energies in those cases, and shows Fig. 8 snapshots of different configurations from the MD simulations. These snapshots show that the alginate oligomer can distort its structure and wrap around the chitosan oligomer in order to better align charged groups. This effect is more pronounced in low salt concentration systems and for the G-oligomer. To further compare these distortions, Fig. 10 shows the root mean squared displacement (RMSD) for the alginate–chitosan system, compared to the geometry optimized vacuum structures. This figure shows that the largest deviation from the vacuum structure occurs for the G form of the alginate, while the values are comparable for the two other forms.

Interestingly, within the scope of the simulated salt concentrations, MG-oligomers were the only ones showing an increase in interaction (lower interaction energy) with an increase in salt concentration, while M- and G-homooligomers showed a decrease. This hints towards an additional stabilization of the chitosan-MG-junction zones through hydrophobic interactions.^68,69^

**Scheme 1:**
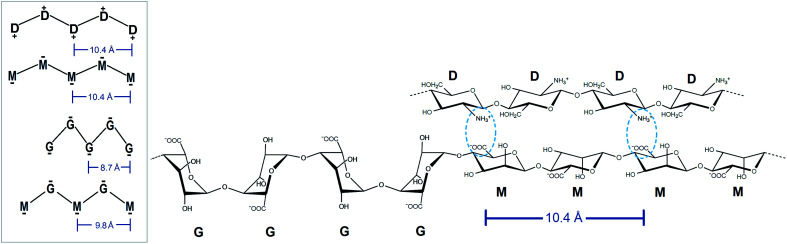
Schematic illustration of the intramolecular charge distances along each side of the used poly- and oligosaccharide chains. Ionic interactions of potentially aligned charged de-*N*-acetylated chitosan and a charged alginate M-block indicated (light blue), allowing for a zipper-like chain alignment. Monosaccharide units: glucosamine (D), mannuronate (M), guluronate (G).

**Fig. 11:**
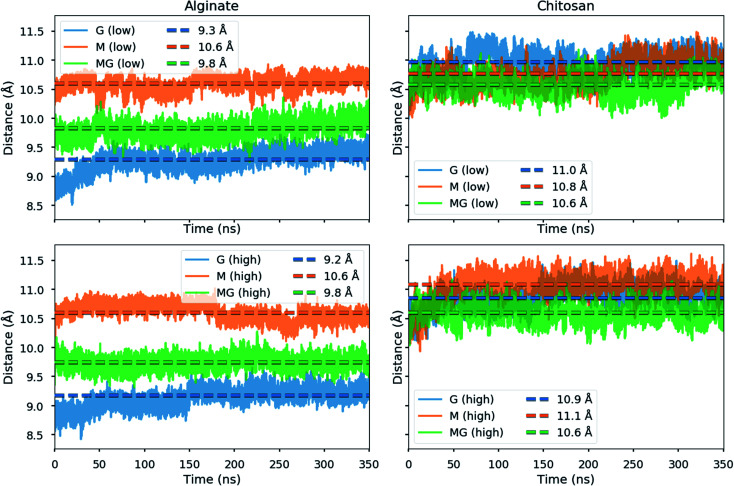
Intramolecular distances between alternating carboxyl groups (left) or amino groups (right) in alginate and chitosan oligomers from the MD simulations of the sandwiched 2 : 1 alginate : chitosan systems.

Due to the distortion of the molecular structures (as shown in Fig. 8 and 10), the charge distances given in Scheme 1 may change. These charge distances were monitored in the MD simulations by calculating the 3D charge distances for every second carboxyl group or amino group along the polysaccharide chains (see Fig. 11 which show these distances for the sandwiched 2 : 1 alginate : chitosan system). An overlap in charge distance between poly-M alginate and chitosan was previously hypothesized to establish a zipper-like junction zone between two polymer chains along the opposing sides of a chitosan oligomer (see Scheme 1 and ESI†).^36^ The calculated charge distances, anticipating freely moving molecules in vacuum, were compared with previous measurements obtained from X-ray crystallography.^38–40^

The simulated oligomers showed a varying degree of good agreement for the calculated charge distances *versus* distances obtained from crystal diffraction (see Table 2). For chitosan and poly-M the measured values and calculated values are almost identical. However, we note that the values for chitosan span a small range (10.6–11.1 Å) when solvated in water. In general, the simulated structures have a larger range of possible stable conformations when solvated in water, and this is reflected in the calculated distances. In particular, the carboxyl groups at the ends of the alginate oligomers exhibit some more freedom in the simulations with water compared to the vacuum structure. For poly-G the simulation delivered a charge distance 6.9% longer than the one obtained from the crystal structures (8.7 *versus* 9.3 Å). However, it should be considered that polysaccharide crystal structures are often stabilized by intermolecular hydrogen bonds, which can lead to slight distortions of atomic bond angles and dihedral angles. Those distortions will not occur for isolated molecules in vacuum or may average out in solution.

**Table 2:** Polymer charge distance for every second carboxyl or amino group along the polysaccharide chain. Comparison of previously determined charge distances through crystal structure analysis *versus* calculated distances for the MD simulations.^38–40^

Polymer	Crystal (Å)	MD simulations in water (Å)
Chitosan	10.4	10.6–11.1
Poly-M	10.4	10.6
Poly-MG	—	9.8
Poly-G	8.7	9.2–9.3

The calculated intramolecular charge distance for poly-MG (9.8 Å) is intermediate of the values determined for poly-M (10.6 Å) and poly-G (9.3 Å) and still relatively close to the value for chitosan (10.6–11.1 Å). This relative overlap in interval distances of poly-MG and chitosan might explain why longer chitosan oligomers are able to form stable hydrogel scaffolds, like poly-M, while CHOS applied to poly-guluronate just leads to a precipitation. Interestingly, we see that the strongest interactions are not obtained for systems where the calculated charge distances in alginate and chitosan are “best” matched. This can be explained by the observed distortions in the structures (see Fig. 8) which may lead to alignment of charged groups.

**Fig. 12:**
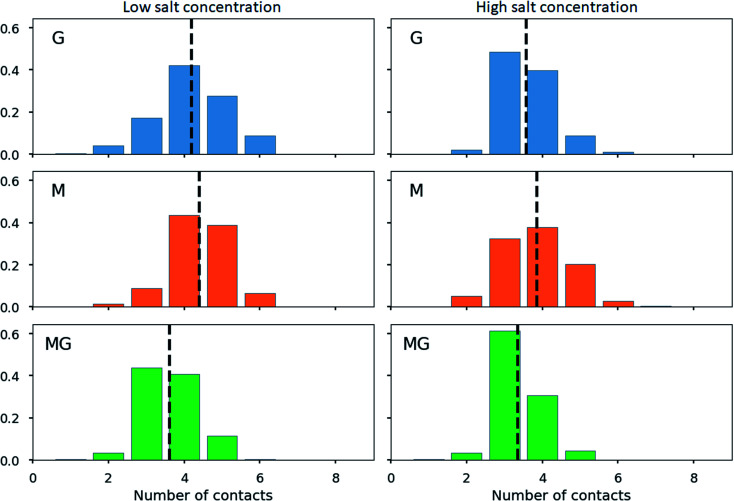
Distribution of number of contacts between COO^−^ groups in an alginate oligomer (DP = 12) and NH_3_^+^ groups in a chitosan oligomer (DP = 8). A contact is defined as a COO^−^ group in alginate being within 3 Å of any NH_3_^+^ group in chitosan. The dashed vertical lines show the average number of contacts in each case. The figure show results for a low (left) and a high (right) concentration of ions (Na^+^/Cl^−^).

To further characterize the chitosan alginate junction zone, the number of contacts between carboxyl and amino groups for various compositions of chitosan and alginate oligomers has been determined. Hereby, contact refers to a distance criterion of ≤3 Å in between any NH_3_^+^ with any COO^−^ group. For the chitosan octamer interacting with a single dodecamer of each type of alginate (see Fig. 12), the average number of contacts for the M-, and G-alginate was slightly above 4, while being slightly below 4 for the MG-alginate. The average number of contacts was reduced at high salt concentration for all three types of alginate.

**Fig. 13:**
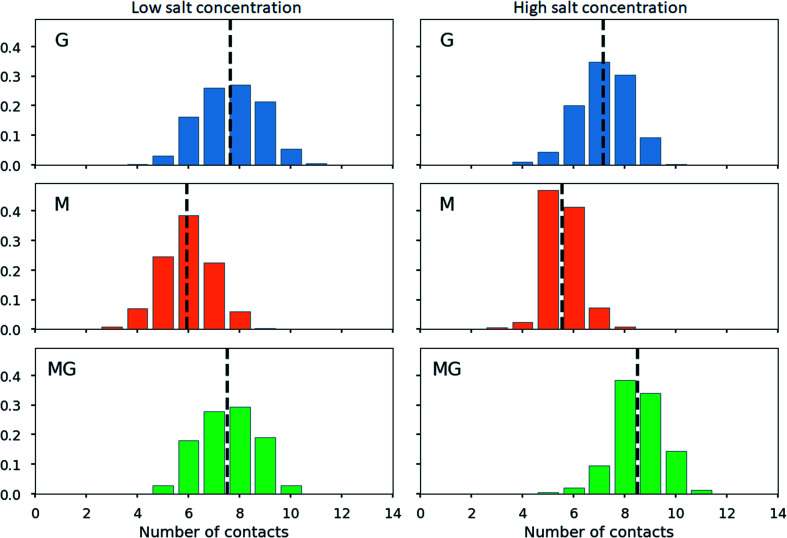
Distribution of number of contacts between COO^−^ groups in alginate oligomers (DP = 12) and NH_3_^+^ groups in a chitosan oligomer (DP = 8) for 2 : 1 alginate : chitosan mixtures. A contact is defined as a COO^−^ group in alginate being within 3 Å of any NH_3_^+^ group in chitosan. The dashed vertical lines show the average number of contacts in each case. The figure show results for a low (left) and a high (right) concentration of ions (Na^+^/Cl^−^).

The calculation of contact points for alginate and chitosan oligomers on a 2 : 1 ratio revealed an average slightly below 8 (see Fig. 13), for G- and MG-segments ionically crosslinked with the chitosan octamer (low salt concentration), while showing an average of 7 contacts for the M-alginate complex. When the salt concentration was increased, the number of contacts decreased for M- and G-alginate, while it increased for the MG-segments to an average above 8. A further noteworthy observation, M-alginate showed a maximum of 8 contacts chelating the chitosan octamer, while G- and especially MG-alginate showed a major distribution towards 9 and 10 contact points. A calculation on the type of interactions, comparing the force field contributions for charge-based interactions (Coulomb) with hydrophobic interactions (Lennard-Jones), revealed that the interaction between the chitosan and alginate is dominated by coulombic interactions in all cases (see ESI†).

## 4 Discussion

Poly-MG alginate gel crosslinked with chitosan oligomers showed the ability to form hydrogels which are orders of magnitude stronger than their poly-M counterparts. Further, an increase in chitosan oligomer DP led to a higher relative increase in gel strength for poly-MG compared to poly-M, suggesting a different underlaying crosslinking mechanism for the two different types of alginate block structures.

To investigate the association and binding of chitosan oligomers to the different alginate block structures, a set of circular dichroism experiments was performed, comparing the three alginate types upon the addition of CHOS at various DPs and concentrations.

Circular dichroism has been used in the past as a sensitive and reliable method to investigate the diadic composition of alginates, as well as characterizing the interaction of calcium ions with the three different types of block structure.^42,64^ It was previously demonstrated that the chiroptical properties of carboxyl chromophores, including guluronic and mannuronic acid, strongly depend on the local ring geometry, ^4^C_1_*versus*^1^C_4_ conformation and neighbouring units.^65,66^

Alginate exhibits strong CD bands between 190–230 nm, originating from n → π* transitions of the carboxylic acid group (with some contributions from π → π* transitions below 200 nm).^65,70^

The chain–chain association expressed when G-sequences are ionically connecting through calcium in a chelate type binding, have been previously described using circular dichroism. The interpretation of CD spectral intensity changes as a consequence of G-block dimerization through calcium (“egg-box” formation) are widely accepted.^67^ Morris *et al.* (1975) demonstrated a linear change in band intensity when the concentration of calcium ions was increased at constant poly-guluronate concentration, whereas no change was observed when applied for poly-mannuronate. This clearly indicated a conformational effect on the poly-G chain (secondary structure) induced by Ca^2+^. No similar effect was observed for the poly-M analog. Donati *et al.* (2005) extended this investigation using enzymatically produced strictly alternating poly-MG alginate.^42^ Variations in molar ellipticity, showed an induced conformational effect through Ca^2+^ on MG-blocks similar to poly-G (albeit with smaller intensity differences).

In this study, a similar approach was conducted, comparing chiroptical properties of three different alginate block types upon the addition of stoichiometric amounts of CHOS. Since the CD bands of carboxylic acid groups are influenced by their protonation, the pH of all CD sample solutions was adjusted to 4.5 in advance (strong interaction of alginate and CHOS previously determined).^65,70^ The molar ellipticities of the three different pure alginates and two chitosans were in excellent agreement with earlier reported data.^64,71^

The addition of chitosan tetramers to poly-M at half the alginate concentration, lead to an elevated positive peak at 199 nm and a deepened negative ellipticity with a minimum at 220 nm. The observed CD change aligns with the concept of “zipper like” junction zones between chitosan and mannuronan, proposed by Khong *et al.* (2013) due to their match in charge distance. The structural effect induced by the chitosan tetramer at half the poly-M concentration, does not further increase at equal and double concentration of tetramer to alginate, suggesting a sandwich like conjugation of chitosan in between two poly-M chains. When the chitosan octamer is applied, the effect at half the alginate concentration overlaps with the effect induced by CHOS DP = 4. However, unlike for the tetramer, the gain in molar ellipticity doubles when the CHOS DP = 8 concentration is increased to equal concentration of alginate.

A possible interpretation could be a more rigid conformational change by the chitosan octamer due to less flexibility along the 2/1 helical alginate chain. More likely however, is that the chitosan octamer is able to form more stable associations with isolated alginate segments, due to its four charges per side, ionically stabilizing the association instead of two charges per side for the tetramer.

The fact that the induced conformational effect further increases when the octamer concentration is increased to double the alginate concentration, gives more validity to this hypothesis. When the chitosan DP = 8 concentration is twice the alginate concentration, a further stabilization of the alginate chain might occur by CHOS associating from both sides of the alginate chain.

None of these induced structural effects, detected by CD spectroscopy, was observed for poly-G or poly-MG. Additionally, an equal concentration of CHOS lead to precipitation of the polysaccharide aggregates for both poly-G and poly-MG. The CD results for poly-M provide further evidence for the “zipper like” gelling concept, where probably a match in charge distance between chitosan and mannuronan, leads to a conformational change in the polymer chain and to the formation of a higher ordered structures.^36,72^ While none of these induced structural effects was observed for MG-blocks, the poly-MG-CHOS gelling systems still displays high gel strengths during rheological experiments, orders of magnitude above their poly-M analogue (when chitosan DP > 6). This suggest a fundamentally different gelling mechanism, underlaying the formation of chitosan-poly-MG junction zones.

A clue to the different underlaying gelling mechanism might be taken from the visual appearance of the hydrogels. While poly-M-CHOS gels are clear and almost fully transparent, poly-MG-CHOS gels were visually observed to be highly turbid, indicative of a possible phase separation.^68^

According to gel theory, ionically crosslinked hydrogels are weakened upon the addition of salt. The salt ions are shielding off attractive electrostatic forces of the ionic crosslinker and reduce the entropic driving force for electrostatic interaction, leading to a decrease in gel strength and gelling kinetics, potentially even to the collapse of gel network or precipitation.^73^ If the junction zone, however, is stabilised through a phase separation, the addition of salt increases the difference in polarity between the solvent and junction zone and is further stabilising the association.

Nowak *et al.* (2003) demonstrated for amphiphilic polypeptide hydrogels, containing hydrophilic charged segments of poly-l-lysine and poly-l-glutamic acid as well as hydrophobic segments of poly-l-leucine, that the hydrogels (at 3.0 wt%) are increasing in gel strength when 50 mM NaCl is added to the system, up until 250 mM NaCl. Simultaneously an increasing turbidity was observed. For amphiphilic polypeptide hydrogels at 1.0 wt% (or lower), a weakening of the gels and subsequent precipitation upon the addition of salt was detected. The combination of hydrophobic and hydrophilic segments for the hydrogels showed a unique tolerance towards different pH and salt conditions, attributed to self-assembly of hydrophobic R-helical domains.^68,74^ A similar approach was conducted by Boisseson *et al.* (2004), who modified alginates covalently linking long hydrophobic alkanes (octadecyl and dodecyl chains) to the polysaccharide backbone. The hydrogels beads were formed through ionotropic gelation using the alginate dropping method into calcium solution. The addition NaCl in the CaCl_2_ solution increased the stability of the calcium alginate hydrogels due to increasing hydrophobic interactions of the alkyl side chains and reduced swelling of the beads.^69^

To investigate a stabilization through a phase separation and potentially the involvement of hydrophobic interactions stabilizing the poly-MG-CHOS junction zones, rheological experiments have been conducted analyzing poly-M and poly-MG crosslinked with a pure chitosan octamer upon the addition of 0, 50, 150, 500 mM NaCl. The transparent poly-M-chitosan gel followed the trend of a typical ionic tropic gelation, displaying reduced overall gel strength and slower gelling kinetics with an increasing salt concentration. Further supporting the zipper-like gelling concept of the two biopolymers. The turbid poly-MG chitosan octamer gelling system on the other hand, showed a sharp increase in gel strength (approx. doubling of *G*′) when 50 mM NaCl was introduced. Furthermore, no deceleration of the kinetics was observed. Unlike poly-M, the alternating MG-blocks were able to form a hydrogel at the highest applied salt concentration of 500 mM. Those results suggest the stabilization of junction zones either through hydrophobic interactions between chitosan and alginates MG-blocks or through a phase separation based on a more efficient compensation of charges in the chelate like complex, creating a localized segment with low polarity. This, to our knowledge, not been reported before.

The rheological results upon the addition of NaCl, as well as the appearance are aligning with previously reported studies on phase separations within hydrogels.^68,75,76^

To gain further inside into the formation of junction zones, chain alignments and binding energies, computer simulations of alginate block oligomers (DP = 12 and 24) and chitosan oligomers (DP = 4, 8) were performed. The interactions of alginate and chitosan at different concentration, ratio, DP, and salt concentration have been calculated. Within the scope of the simulations, no particular conformation of CHOS and alginate strongly differed from the other alginate block types and associations, nor did the binding energies reveal large differences between binding energies and junction zones. Nevertheless, the strongest interaction for a sandwich type chelation (junction zone) was observed for the chitosan octamer chelated in between two MG-blocks at high salt concentration. Further was the chitosan-MG-complex, the only simulated chain association where the binding energy increased upon the addition of salt instead of decreased suggesting an additional stabilization of the junction zone by hydrophobic interactions, not present in poly-M-chitosan-hydrogels. Calculations on the coulomb and Lennard-Jones potential revealed only small contributions of hydrophobic interactions.

When calculating the number of contacts between the chitosan octamer and the different alginates, the contact number for M-alginate was close to but never exceeded 8 (the number of available amino groups), giving further evidence to the previously hypothesised zipper-like chain alignment of these to poly- and oligosaccharides. For the chitosan octamer chelated by MG-alginate the number of contacts was close to 8 but with a significant distribution towards 9 contacts. Upon the addition of salt, those values further increased, showing an average number of contacts above 8 with even a significant distribution at 10 contacts.

The high number of contacts indicates a very compact junction zone formation for CHOS-poly-MG. While the data for poly-M chitosan junction zones clearly indicates a gel formation based on poly-ionic interactions, for MG-alginate we suggest (at least) two different interaction mechanism working simultaneously: electrostatic and hydrophobic.

The calculated distance of consecutive charges along MG-blocks (9.8 Å) will be close enough to chitosan (10.5 Å) to initiate a Manning condensation between the two block counterions and electrostatic interactions will be the predominant factor for the formation of chitosan poly-MG junction zones.^77^ However, it was demonstrated through molecular dynamics that hydrophobic interactions (Lennard-Jones) still contribute to some extent. At various ionic strengths, the different processes would contribute to the formation of junction zones to a different extent, which is observed both at the increase in *G*′ as well as the delay in gelling kinetics upon the addition of NaCl to the CHOS-poly-MG gelling system.

The CHOS poly-MG hydrogel might be further stabilized through a precipitation or local phase separation effect along the junction zone. Although this effect remains speculative, the increasing turbidity during the poly-MG chitosan hydrogel formation as well as the gel strength with magnitudes of order greater than for the equivalent poly-M gel supports this hypothesis.

Alternatively, the increasing turbidity with increasing gelling time could be attributed to a formations of higher order (random) structures as seen with (*e.g.*) alginic acid gels described through SAXS experiments.^78^ Also an additional formation of hydrogen bonds within the poly-MG chitosan junction zone has to be considered.^38,79^

## 5 Conclusion

Strong evidences have been obtained, supporting the previously proposed gelling principle of poly-M alginate crosslinked with chitosan oligomers due to a match in charge distance between the two polyelectrolytes.^36^ CD spectroscopy revealed an increased chiroptical activity for poly-M chitosan gelling systems, indictive of induced conformational changes and formation of higher ordered structures.^42,80^

A unique chitosan oligomer poly-MG gelling system was described using circular dichroism beside rheology and computer simulations. Rheological measurement revealed gel strengths magnitudes of order higher than displayed for its poly-M analogue. Furthermore, a strong increase in gel strength upon the addition of 50 and even 150 mM NaCl for the chitosan and poly-MG gelling system was detected, suggesting a stabilization of the junction zones through hydrophobic interactions and/or a phase separation, which to our knowledge has not been reported before. Those findings were supported by molecular dynamics simulations, showing an increase in interaction energies between chitosan and alginate upon the addition of NaCl for poly-MG, while for poly-M and poly-G the addition of NaCl decreased the interaction energies with chitosan. CD measurements showed no systematic alignment or induced conformational change within the poly-MG chitosan system, which further implies a fundamentally different effect supporting the chitosan poly-MG junction zones. The high gel strengths, especially upon the addition of salt, as well as the turbidity of the poly-MG chitosan gelling system are indictive of a more complex formation of junction zones within these hydrogels, than seen within comparable ionically crosslinked alginate gelling systems.^68^

The described CHOS poly-MG gelling systems are displaying high gel strengths, are known to be fully biocompatible and have revealed a high tolerance to a broad range of salt concentrations present in various biological systems, which might prove poly-MG chitosan gels highly relevant for biomedical applications.

## Conflicts of interest

There are no conflicts to declare.

## Supplementary Material

RA-011-D1RA01003D-s001

## References

[cit1] Painter T. J. , in The Polysaccharides, ed. G. O. Aspinall, 1983, pp. 195–285

[cit2] Linker A., Jones R. S. (1966). A new polysaccharide resembling alginic acid isolated from pseudomonads. J. Biol. Chem..

[cit3] Gorin P. A. J., Spencer J. F. T. (1966). Exocellular aginic acid from Azotobacter vinelandii. Can. J. Chem..

[cit4] Draget K. I. , Moe S., Skjåk-Bræk G. and Smidsrød O., Food Polysaccharides and Their Applications, 2nd edn, CRC Press, Taylor & Francis Group, 2006, pp. 290–328, ISBN: 0-8247-5922-2

[cit5] Fukushima M., Tatsumi K., Wada S. (1999). Evaluation of the Intrinsic Acid-Dissociation Constant of Alginic Acid by Considering the Electrostatic Effect. Anal. Sci..

[cit6] Vårum K. M. and Smidsrød O., in Polysaccharides - Structural Diversity and Functional Versatility, ed. S. Dumitriu, CRC Press, Boca Raton, 2nd edn, 2005, pp. 625–642

[cit7] Anthonsen M. W., Smidsrød O. (1995). Hydrogen ion titration of chitosans with varying degrees of N-acetylation by monitoring induced 1H-NMR chemical shifts. Carbohydr. Polym..

[cit8] Strand S. P., Tømmeraas K., Vårum K. M., Østgaard K. (2001). Electrophoretic Light Scattering Studies of Chitosans with Different Degrees of N -acetylation. Biomacromolecules.

[cit9] Tsukada S., Inoue Y. (1981). Conformational properties of chito-oligosaccharides: titration, optical rotation, and carbon-^13^NMR. studies of chito-oligosaccharides. Carbohydr. Res..

[cit10] Vårum K. M., Ottøy M. H., Smidsrød O. (1994). Water-solubility of partially N-acetylated chitosans as a function of pH: effect of chemical composition and depolymerisation. Carbohydr. Polym..

[cit11] Aam B. B., Heggset E. B., Norberg A. L., Sørlie M., Vårum K. M., Eijsink V. G. H. (2010). Production of chitooligosaccharides and their potential applications in medicine. Mar. Drugs.

[cit12] Nilsen-Nygaard J., Strand S., Vårum K., Draget K., Nordgård C. (2015). Chitosan: Gels and Interfacial Properties. Polymers.

[cit13] Sacco P., Furlani F., de Marzo G., Marsich E., Paoletti S., Donati I. (2018). Concepts for Developing Physical Gels of Chitosan and of Chitosan Derivatives. Gels.

[cit14] Furlani F., Sacco P., Scognamiglio F., Asaro F., Travan A., Borgogna M., Marsich E., Cok M., Paoletti S., Donati I. (2019). Nucleation, reorganization and disassembly of an active network from lactose-modified chitosan mimicking biological matrices. Carbohydr. Polym..

[cit15] Nordtveit R. J., Vårum K. M., Smidsrød O. (1996). Degradation of partially N-acetylated chitosans with hen egg white and human lysozyme. Carbohydr. Polym..

[cit16] Vårum K. M., Myhr M. M., Hjerde R. J. N., Smidsrød O. (1997). *In vitro* degradation rates of partially N-acetylated chitosans in human serum. Carbohydr. Res..

[cit17] Köping-Höggård M., Tubulekas I., Guan H., Edwards K., Nilsson M., Vårum K. M., Artursson P. (2001). Chitosan as a nonviral gene delivery system. Structure-property relationships and characteristics compared with polyethylenimine *in vitro* and after lung administration in vivo. Gene Ther..

[cit18] Strand S., Danielsen S., Christensen B. E., Vårum K. M. (2005). Influence of Chitosan Structure on the Formation and Stability of DNA- Chitosan Polyelectrolyte Complexes. Biomacromolecules.

[cit19] Dutta P. K., Tripathi S., Mehrotra G. K., Dutta J. (2009). Perspectives for chitosan based antimicrobial films in food applications. Food Chem..

[cit20] Felt O., Carrel A., Baehni P., Buri P., Gurny R. (2000). Chitosan as tear substitute: a wetting agent endowed with antimicrobial efficacy. J. Ocul. Pharmacol. Ther..

[cit21] Liu X. F., Guan Y. L., Yang D. Z., Li Z., Yao K. D. (2000). Antibacterial Action of Chitosan and Carboxymethlyated Chitosan. J. Appl. Polym. Sci..

[cit22] Mellegård H., Strand S. P., Christensen B. E., Granum P. E., Hardy S. P. (2011). Antibacterial activity of chemically defined chitosans: Influence of molecular weight, degree of acetylation and test organism. Int. J. Food Microbiol..

[cit23] Xia W., Liu P., Zhang J., Chen J. (2011). Biological activities of chitosan and chitooligosaccharides. Food Hydrocolloids.

[cit24] Ong S. Y., Wu J., Moochhala S. M., Tan M. H., Lu J. (2008). Development of a chitosan-based wound dressing with improved hemostatic and antimicrobial properties. Biomaterials.

[cit25] Ueno H., Mori T., Fujinaga T. (2001). Topical formulations and wound healing applications of chitosan. Adv. Drug Delivery Rev..

[cit26] Ross-Murphy S. B. , in Biophysical Methods in Food Research, ed. H. W. S. Chan, Blackwell Scientific Publications, 1984, pp. 137–199 10.1016/0309-1740(84)90060-3 22055003

[cit27] Mi F. L., Shyu S. S., Wong T. B., Jang S. F., Lee S. T., Lu K. T. (1999). Chitosan-polyelectrolyte complexation for the preparation of gel beads and controlled release of anticancer drug. II. Effect of pH-dependent ionic crosslinking or interpolymer complex using tripolyphosphate or polyphosphate as reagent. J. Appl. Polym. Sci..

[cit28] Desai R. M., Koshy S. T., Hilderbrand S. a., Mooney D. J., Joshi N. S. (2015). Versatile click alginate hydrogels crosslinked via tetrazine-norbornene chemistry. Biomaterials.

[cit29] Li H. (2010). Preparation and Characterization of Homogeneous Hydroxyapatite/Chitosan Composite Scaffolds via In-Situ Hydration. J. Biomater. Nanobiotechnol..

[cit30] Guan Y. L., Shao L. E. I., De Yao K. (1996). A Study on Correlation Between Water State and Swelling Kinetics of Chitosan-Based Hydrogels. J. Appl. Polym. Sci..

[cit31] Draget K. I. and Skjåk-Bræk G., Renewable Resources for Functional Polymers and Biomaterials: Polysaccharides, Proteins and Polyesters, 2011, pp. 186–209, ISBN: 978-1-84973-245-1

[cit32] Leong J.-Y., Lam W.-H., Ho K.-W., Voo W.-P., Lee M. F.-X., Lim H.-P., Lim S.-L., Tey B.-T., Poncelet D., Chan E.-S. (2015). Advances in fabricating spherical alginate hydrogels with controlled particle designs by ionotropic gelation as encapsulation systems. Particuology.

[cit33] Smidsrød O., Skjåk-Bræk G. (1990). Alginate as immobilization matrix for cells. Minerva Biotecnol..

[cit34] Mørch Ý., Donati I., Strand B. (2006). Effect of Ca^2+^, Ba^2+^, and Sr^2+^ on Alginate Microbeads. Biomacromolecules.

[cit35] Sikorski P., Mo F., Skjåk-Bræk G., Stokke B. T. (2007). Evidence for egg-box-compatible interactions in calcium - Alginate gels from fiber x-ray diffraction. Biomacromolecules.

[cit36] Khong T. T., Aarstad O. A., Skjåk-Bræk G., Draget K. I., Vårum K. M. (2013). Gelling concept combining chitosan and alginate - Proof of principle. Biomacromolecules.

[cit37] Feng Y., Kopplin G., Sato K., Draget K. I., Vårum K. M. (2017). Alginate gels with a combination of calcium and chitosan oligomer mixtures as crosslinkers. Carbohydr. Polym..

[cit38] Minke R., Blackwell J. (1978). The structure of α-chitin. J. Mol. Biol..

[cit39] Sørbotten A., Horn S. J., Eijsink V. G. H., Vårum K. M. (2005). Degradation of chitosans with chitinase B from Serratia marcescens. FEBS J..

[cit40] Atkins E. D., Mackie W., Smolko E. E. (1970). Crystalline structures of alginic acids. Nature.

[cit41] Høidal H. K., Ertesvåg H., Skjåk-Bræk G., Stokke B. T., Valla S. (1999). The recombinant Azotobacter vinelandii mannuronan C-5-epimerase AlgE4 epimerizes alginate by a nonrandom attack mechanism. J. Biol. Chem..

[cit42] Donati I., Holtan S., Mørch Y. A., Borgogna M., Dentini M., Skjåk-Bræk G. (2005). New hypothesis on the role of alternating sequences in calcium-alginate gels. Biomacromolecules.

[cit43] Holtan S., Zhang Q., Strand W. I., Skjåk-Bræk G. (2006). Characterization of the Hydrolysis Mechanism of Polyalternating Alginate in Weak Acid and Assignment of the Resulting MG-Oligosaccharides by NMR Spectroscopy and ESI−Mass Spectrometry. Biomacromolecules.

[cit44] Arlov Ø., Aachmann F. L., Sundan A., Espevik T., Skjåk-Bræk G. (2014). Heparin-like properties of sulfated alginates with defined sequences and sulfation degrees. Biomacromolecules.

[cit45] Aarstad O. A., Tøndervik A., Sletta H., Skjåk-Bræk G. (2012). Alginate sequencing: An analysis of block distribution in alginates using specific alginate degrading enzymes. Biomacromolecules.

[cit46] Páll S., Schulz R., Hess B., Smith J. C., Murtola T., Abraham M. J., Lindahl E. (2015). GROMACS: High performance molecular simulations through multi-level parallelism from laptops to supercomputers. SoftwareX.

[cit47] Schrödinger L. , Schrödinger Release 2018-1: Maestro, Schrödinger, LLC, New York, NY, 2018

[cit48] Bochevarov A. D., Harder E., Hughes T. F., Greenwood J. R., Braden D. A., Philipp D. M., Rinaldo D., Halls M. D., Zhang J., Friesner R. A. (2013). Jaguar: A high-performance quantum chemistry software program with strengths in life and materials sciences. Int. J. Quantum Chem..

[cit49] Shivakumar D., Williams J., Wu Y., Damm W., Shelley J., Sherman W. (2010). Prediction of absolute solvation free energies using molecular dynamics free energy perturbation and the opls force field. J. Chem. Theory Comput..

[cit50] Essmann U., Perera L., Berkowitz M. L., Darden T., Lee H., Pedersen L. G. (1995). A smooth particle mesh Ewald method. J. Chem. Phys..

[cit51] Hess B., Bekker H., Berendsen H. J. C., Fraaije J. G. E. M. (1997). LINCS: A linear constraint solver for molecular simulations. J. Comput. Chem..

[cit52] Mahoney M. W., Jorgensen W. L. (2000). A five-site model for liquid water and the reproduction of the density anomaly by rigid, nonpolarizable potential functions. J. Chem. Phys..

[cit53] Bussi G., Donadio D., Parrinello M. (2007). Canonical sampling through velocity rescaling. J. Chem. Phys..

[cit54] Berendsen H. J. C., Postma J. P. M., Van Gunsteren W. F., Dinola A., Haak J. R. (1984). Molecular dynamics with coupling to an external bath. J. Chem. Phys..

[cit55] Parrinello M., Rahman A. (1981). Polymorphic transitions in single crystals: A new molecular dynamics method. J. Appl. Phys..

[cit56] Grasdalen H., Larsen B., Smidsrød O. (1981). ^13^C NMR Studies of Monomeric Coposition and Sequence in Alginate. Carbohydr. Res..

[cit57] Grasdalen H. (1983). High-field, ^1^H-NMR spectroscopy of alginate: sequential structure and linkage conformations. Carbohydr. Res..

[cit58] Draget K. I., Skjakbrak G., Smidsrod O., Bræk G. S., Smidsrød O. (1994). Alginic acid gels: the effect of alginate chemical composition and molecular weight. Carbohydr. Polym..

[cit59] Draget K. I. , Simensen M. K., Onsøyen E. and Smidsrød O., Fourteenth International Seaweed Symposium, Springer, Netherlands, 1993, pp. 563–569

[cit60] Wang X., Spencer H. G. (1998). Calcium alginate gels: Formation and stability in the presence of an inert electrolyte. Polymer.

[cit61] Smidsrod O., Haug A. (1972). Dependence upon the gel-sol state of the ion-exchange properties of alginates. Acta Chem. Scand..

[cit62] Tanaka T. (1981). Gels. Sci. Am..

[cit63] Gåserød O., Sannes A., Skjåk-Bræk G. (1999). Microcapsules of alginate-chitosan. II. A study of capsule stability and permeability. Biomaterials.

[cit64] Donati I., Gamini A., Skjåk-Bræk G., Vetere A., Campa C., Coslovi A., Paoletti S. (2003). Determination of the diadic composition of alginate by means of circular dichroism: A fast and accurate improved method. Carbohydr. Res..

[cit65] Morris E. R., Rees D. A., Sanderson G. R., Thom D. (1975). Conformation and circular dichroism of uronic acid residues in glycosides and polysaccharides. J. Chem. Soc., Perkin Trans. 2.

[cit66] Morris E. R., Rees D. A., Thom D. (1980). Characterisation of alginate composition and block-structure by circular dichroism. Carbohydr. Res..

[cit67] Morris E. R., Rees D. A., Thom D., Boyd J. (1978). Chiroptical and stoichiometric evidence of a specific, primary dimerisation process in alginate gelation. Carbohydr. Res..

[cit68] Nowak A. P., Breedveld V., Pine D. J., Deming T. J. (2003). Unusual Salt Stability in Highly Charged Diblock Co-polypeptide Hydrogels. J. Am. Chem. Soc..

[cit69] De Boisseson M. R., Leonard M., Hubert P., Marchal P., Stequert A., Castel C., Favre E., Dellacherie E. (2004). Physical alginate hydrogels based on hydrophobic or dual hydrophobic/ionic interactions: Bead formation, structure, and stability. J. Colloid Interface Sci..

[cit70] Listowsky I., Avigad G., Englard S. (1970). Conformational Equilibria and Stereochemical Relationships among Carboxylic Acids. J. Org. Chem..

[cit71] Domard A. (1987). Determination of N-acetyl content in chitosan samples by c.d. measurements. Int. J. Biol. Macromol..

[cit72] Khouri S. J., Knierim R., Buss V. (2009). Induced circular dichroism of the interaction between pinacyanol and algal alginates. Carbohydr. Res..

[cit73] Tang Y., Wang X., Li Y., Lei M., Du Y., Kennedy J. F., Knill C. J. (2010). Production and characterisation of novel injectable chitosan/methylcellulose/salt blend hydrogels with potential application as tissue engineering scaffolds. Carbohydr. Polym..

[cit74] Nowak A. P., Sato J., Breedveld V., Deming T. J. (2006). Hydrogel Formation in Amphiphilic Triblock Copolypeptides. Supramol. Chem..

[cit75] Liu Q., Hedberg E. L., Liu Z., Bahulekar R., Meszlenyi R. K., Mikos A. G. (2000). Preparation of macroporous poly(2-hydroxyethyl methacrylate) hydrogels by enhanced phase separation. Biomaterials.

[cit76] Hua F. J., Do Nam J., Lee D. S. (2001). Preparation of a Macroporous Poly(L-lactide) Scaffold by Liquid-Liquid Phase Separation of a PLLA/1,4-Dioxane/Water Ternary System in the Presence of NaCl. Macromol. Rapid Commun..

[cit77] Manning G. S. (1969). Limiting laws and counterion condensation in polyelectrolyte solutions I. Colligative properties. J. Chem. Phys..

[cit78] Draget K. I., Stokke B. T., Yuguchi Y., Urakawa H., Kajiwara K. (2003). Small-angle X-ray scattering and rheological characterization of alginate gels. 3. Alginic acid gels. Biomacromolecules.

[cit79] Kovalenko V. I. (2010). Crystalline cellulose: structure and hydrogen bonds. Russ. Chem. Rev..

[cit80] Matsuda Y., Biyajima Y., Sato T. (2009). Thermal denaturation, renaturation, and aggregation of a double-helical polysaccharide xanthan in aqueous solution. Polym. J..

