# Supplementary material for: Alginate gels crosslinked with chitosan oligomers – a systematic investigation into alginate block structure and chitosan oligomer interaction
Source: RSC Adv. 2021 Apr 13;11(23):13780–98. doi: 10.1039/d1ra01003d (PMC8697632; doi:10.1039/d1ra01003d)
Supplement: RA-011-D1RA01003D-s001 [file RA-011-D1RA01003D-s001.pdf]

Electronic Supplementary Material (ESI) for RSC Advances.
This journal is © The Royal Society of Chemistry 2021

Supporting Information:

Alginate gels crosslinked with chitosan oligomers — A systematic investigation into alginate block structure and chitosan oligomer interaction

Georg Kopplin[a], Anders Lervik[b], Kurt I. Draget[a] and Finn L. Aachmann*[a]

[a] *Norwegian Biopolymer Laboratory (NOBIPOL), Department of Biotechnology, Norwegian University of Science and Technology, 7491 Trondheim, Norway*
[b] *Department of Chemistry, Norwegian University of Science and Technology, 7491 Trondheim, Norway*

*Corresponding author. E-mail finn.l.aachmann@ntnu.no

Supporting Information

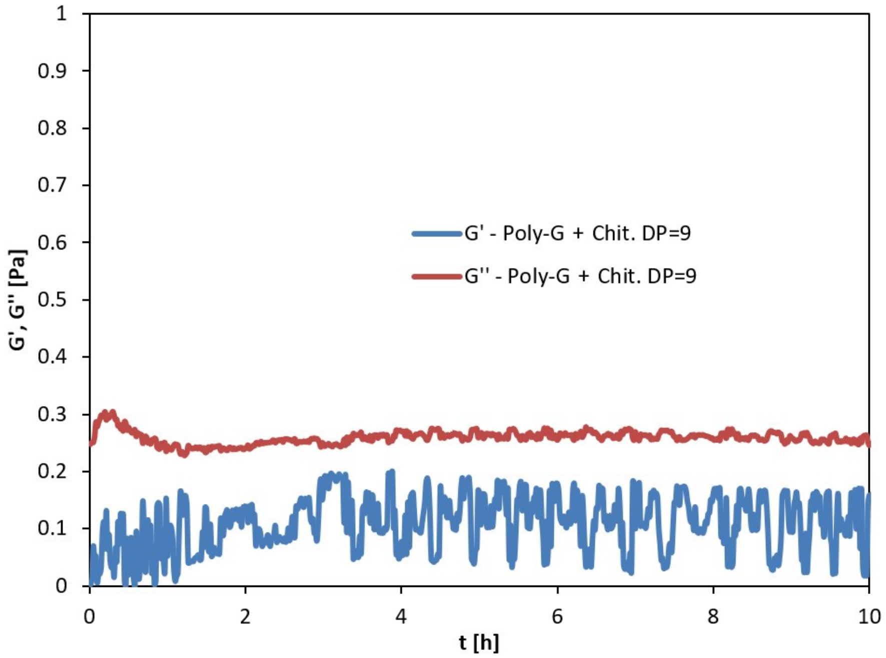

Figure S1: Kinetics of gelation of poly-G alginate concentration at 10 g/L (1%) with chitosan oligomers DP = 9 and concentration at 3 g/L (0.3%). GDL concentration 2 g/L. G' (blue line) and G'' (red line), was determined as a function of time.

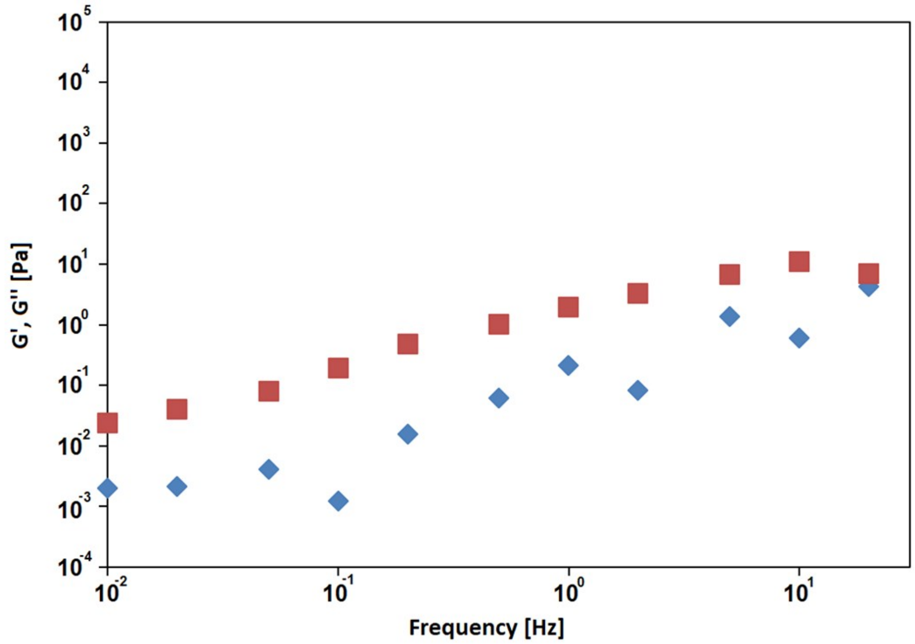

Figure S2: Frequency sweep of poly-MG (1.0%) and CHOS DP = 9 (0.3%), without GDL. G' (blue diamonds), G'' (red squares). All measurements performed at 20°C.

Table S1: Complete composition of alginate hydrogels, used for rheological experiments. Alginate was ionically crosslinked with chitosan oligosaccharides (CHOS) at various CHOS/Alginate monosaccharide unit ratios. The amounts of NaOH was adjusted to reach pH to 8 when mixing CHOS and Alginate. The amount of Glucono δ-lactone (GDL) was adjusted to reach a final pH of 4.35 ± 0.05. For calculations based on the monosaccharide ratio, the molar masses 199.12 g/mol and 197.62 g/mol were assigned to the sodium alginate unit and to the glucosamine chloride unit respectively.

SAMPLE	1	2	3	4	5	Unit
Monosacch. unit ratio: CHOS/Alginate	0.2	0.3	0.4	0.5	0.6	
Alginate solution (30 g/L) added	1.00	1.00	1.00	1.00	1.00	g
Alginate amount	30.00	30.00	30.00	30.00	30.00	mg
Alginate concentration (final)	10.00	10.00	10.00	10.00	10.00	g/L
CHOS amount	6.08	9.12	12.16	15.20	18.24	mg
CHOS solution (100 g/L) added	60.8	91.2	121.6	152.0	182.4	µl
CHOS concentration (final)	2.03	3.04	4.05	5.07	6.08	g/L
NaOH (1 M) added	14.9	22.4	29.8	37.3	44.7	µl
GDL amount	5.32	7.98	10.63	13.29	15.95	mg
GDL concentration (final)	1.77	2.66	3.54	4.43	5.32	g/L
H_2O added (final Vol. 3 mL)	1924	1886	1849	1811	1773	µl

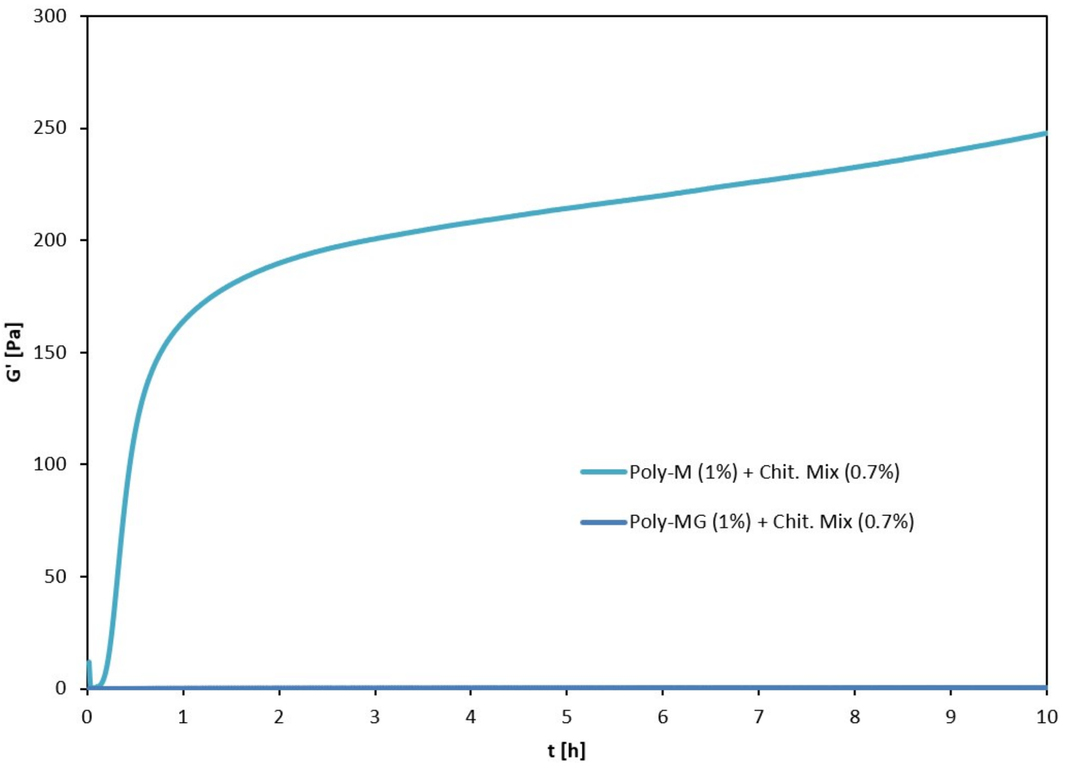

Figure S3: Kinetics of gelation as a function of time. Alginate concentration (poly-M: light blue, poly-MG: dark blue) at 10 g/L (1%). Chitosan oligomer mix (DP_n = 3.96, FA = 0.045) concentration at 7 g/L (0.7%). GDL at 2 g/L. G' was determined as a function of time. G" and delta are not shown in the Figure for reasons of clarity.

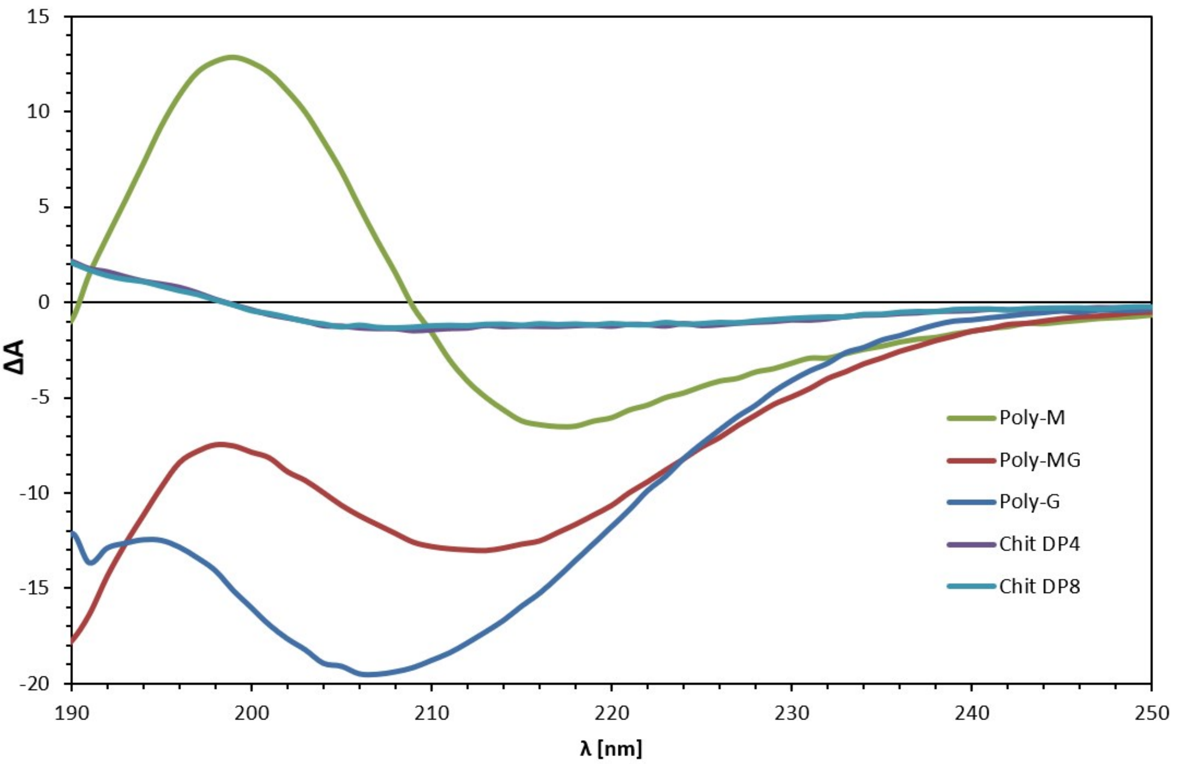

Figure S4: Circular dichroism spectra of poly-M (green), poly-MG (red), poly-G (blue) and chitosan DP = 4 (purple) and DP = 8 (light blue). All measurements were performed at pH = 4.5 and T = 25°C with a sample concentration of 0.4 g/L.

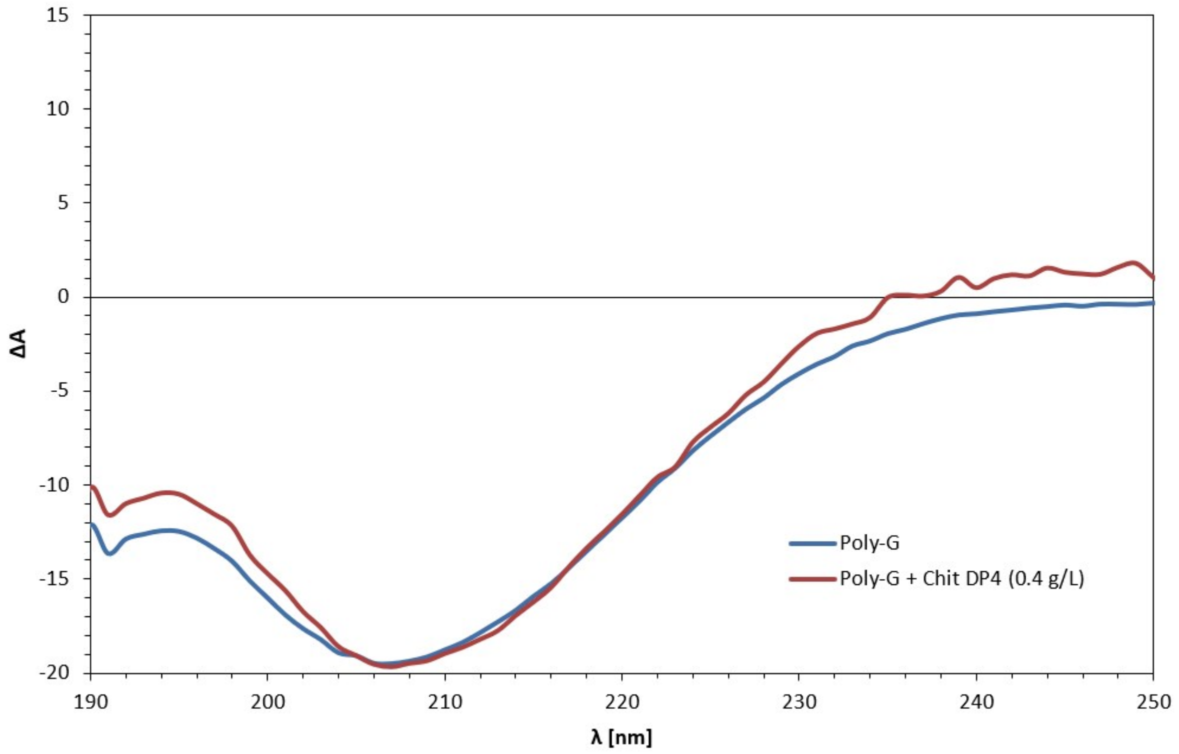

Figure S5: Circular dichroism spectra of poly-G (blue line) and poly-G upon addition of chitosan DP = 4 (red). The alginate and chitosan concentration was at 0.4 g/L. All measurements were performed at pH = 4.5 and T = 25°C.

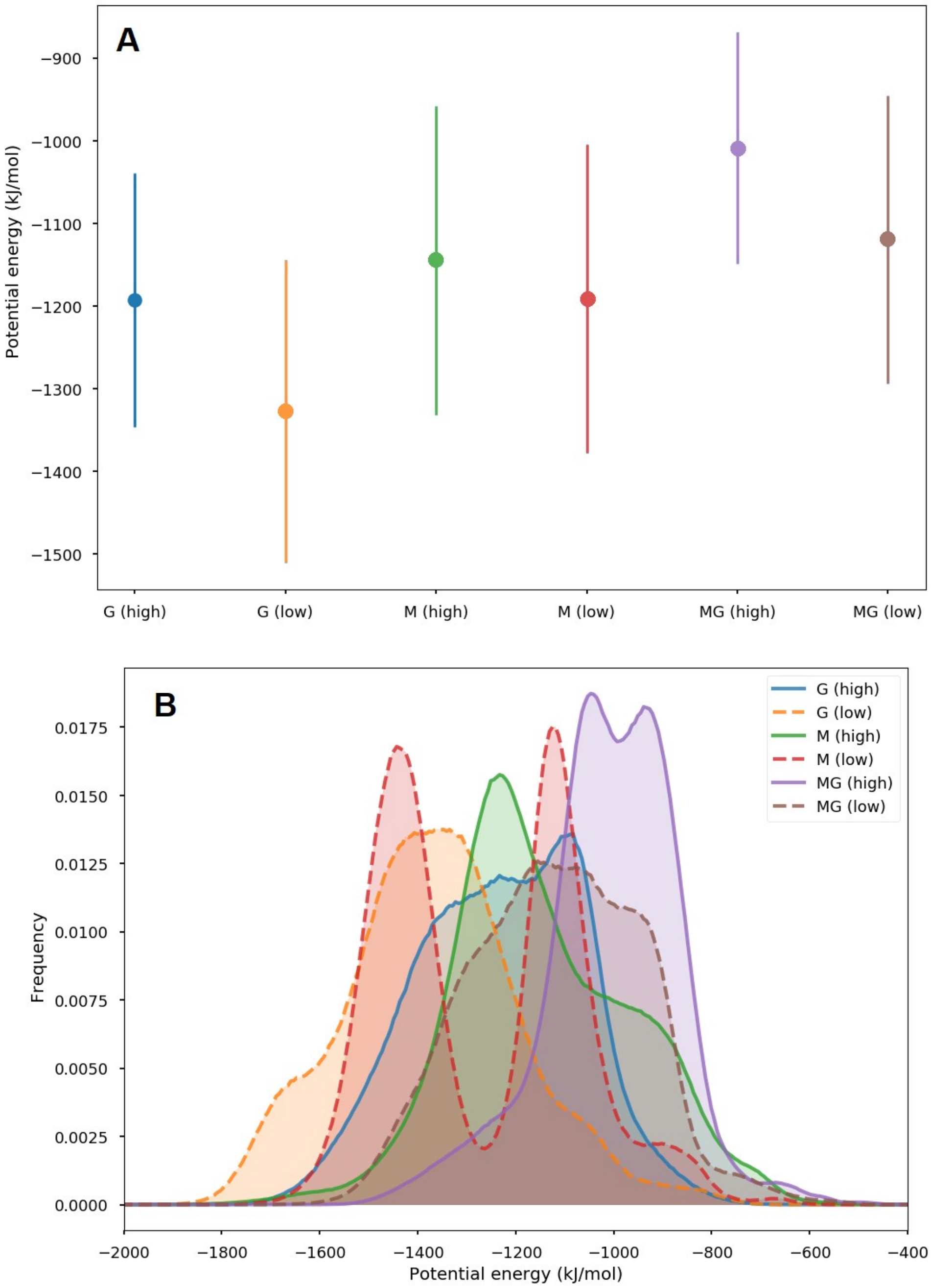

Figure S6: Average (A) and histograms (B) of the interaction energies between alginate oligomers (DP = 12) and a chitosan oligomer (DP = 8), obtained in MD simulations lasting 100 ns. The average interaction energy is lowest for the G oligomer with a low concentration of salt and largest for the MG-oligomer with a high concentration of salt.

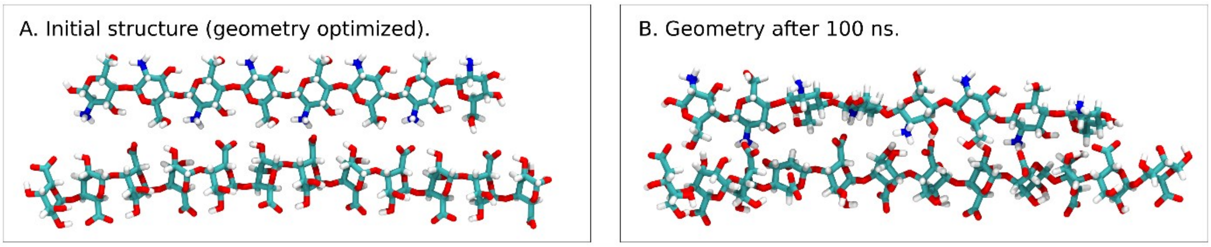

Figure S7: Simulation snapshots of the alginate G-oligomer and a chitosan oligomer (DP = 8) for a low concentration of salt (salt ions are not shown). (Left) The initial geometry optimized structure. (Right) The structure after a MD simulation lasting 100 ns. In this case, distortions of the molecular structures allow for a closer approach of charged groups in the molecules.

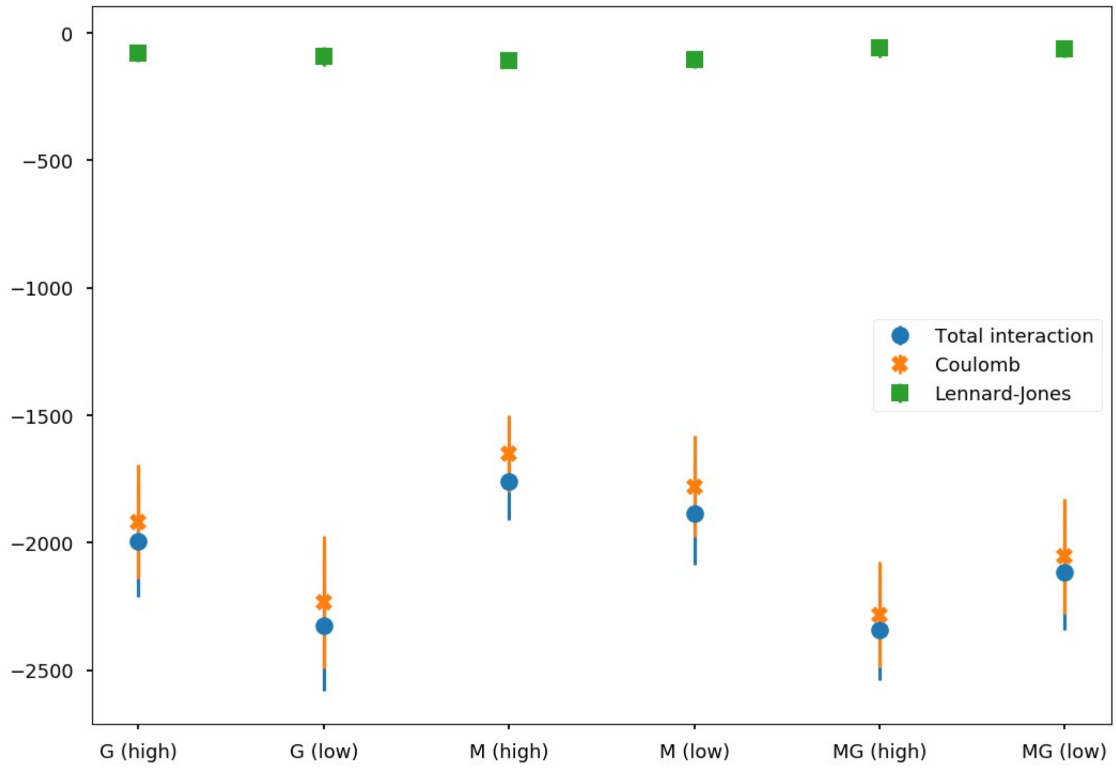

Figure S8: Average interaction energy between alginate (DP = 12) and chitosan (DP = 8) oligomers for 2:1 alginate:chitosan mixtures and high and low ion (Na^+/Cl^-) concentrations.

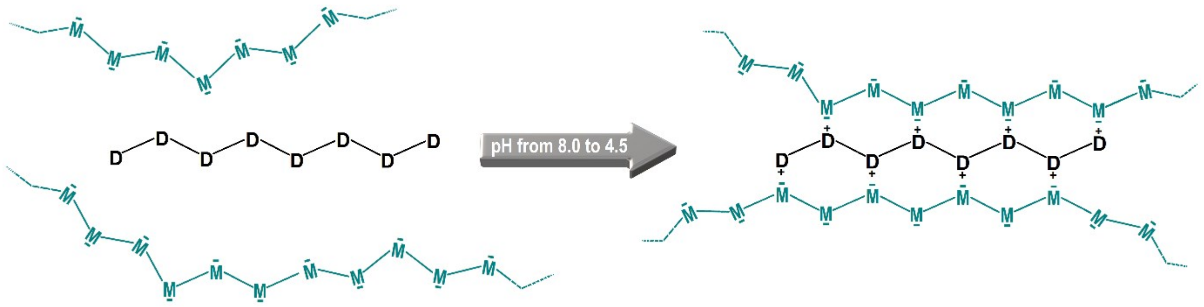

Scheme S1: Schematic illustration of the interaction between a chitosan oligomer (black) and poly-M alginate (green) at different pHs. Monosaccharide units: glucosamine (D), mannuronate (M). At pH ≤ 8 the amino groups of chitosan are mostly uncharged, while the carboxyl groups of alginate are mostly charged. At pH 4.0–5.0 both chitosan and alginate are predominantly charged, leading to ionic crosslinking of the two components and a zippier-like chain alignment due to the similar charge distance in both compounds.
